# Novel subgroups of attention-deficit/hyperactivity disorder identified by topological data analysis and their functional network modular organizations

**DOI:** 10.1371/journal.pone.0182603

**Published:** 2017-08-22

**Authors:** Sunghyon Kyeong, Jae-Jin Kim, Eunjoo Kim

**Affiliations:** 1 Severance Biomedical Science Institute, Yonsei University College of Medicine, Seoul, Republic of Korea; 2 Department of Psychiatry and Institute of Behavioral Science in Medicine, Yonsei University College of Medicine, Seoul, Republic of Korea; Tianjin University, CHINA

## Abstract

Attention-deficit/hyperactivity disorder (ADHD) is a clinically heterogeneous condition and identification of clinically meaningful subgroups would open up a new window for personalized medicine. Thus, we aimed to identify new clinical phenotypes in children and adolescents with ADHD and to investigate whether neuroimaging findings validate the identified phenotypes. Neuroimaging and clinical data from 67 children with ADHD and 62 typically developing controls (TDCs) from the ADHD-200 database were selected. Clinical measures of ADHD symptoms and intelligence quotient (IQ) were used as input features into a topological data analysis (TDA) to identify ADHD subgroups within our sample. As external validators, graph theoretical measures obtained from the functional connectome were compared to address the biological meaningfulness of the identified subtypes. The TDA identified two unique subgroups of ADHD, labelled as mild symptom ADHD (mADHD) and severe symptom ADHD (sADHD). The output topology shape was repeatedly observed in the independent validation dataset. The graph theoretical analysis showed a decrease in the degree centrality and PageRank in the bilateral posterior cingulate cortex in the sADHD group compared with the TDC group. The mADHD group showed similar patterns of intra- and inter-module connectivity to the sADHD group. Relative to the TDC group, the inter-module connectivity between the default mode network and executive control network were significantly increased in the sADHD group but not in the mADHD group. Taken together, our results show that the data-driven TDA is potentially useful in identifying objective and biologically relevant disease phenotypes in children and adolescents with ADHD.

## Introduction

Attention-deficit/hyperactivity disorder (ADHD) is the most common neurodevelopmental disorder of childhood, affecting 5% of school-age children worldwide [1]. ADHD is a clinically heterogeneous condition, with deficits in multiple neuropsychological processes and related brain systems [2]. Thus, different individuals with ADHD display diverse profiles in cognitive, emotional and motivational domains [3]. Therefore, there may be different disease phenotypes under the diagnosis of ADHD, and the identification of subgroups with distinct patterns of clinical manifestation may lead to the development of more effective and targeted intervention for personalized treatment and improved patient care [4].

Recent efforts in data sharing, such as the ADHD-200 Consortium have made it possible to develop a large dataset representative of the whole spectrum of the disorder. The ADHD-200 dataset provides information on age, sex, intellectual ability, symptom severity, and neuroimaging data from hundreds of individuals with ADHD and TDC [5]. To identify clinically useful new subgroups from large datasets, the choice of an appropriate clustering method and input features are important because varying results are expected depending on the machine learning algorithm and input features used [4]. Regarding the clustering method, the classical unsupervised machine learning method, e.g., *k*-means clustering, has been widely used for this purpose. However, a major criticism of this method is that identified subgroups are locally optimized, given that the initial clusters are heuristically assigned. Furthermore, the *k*-means clustering would fail to detect subgroups if the shape of the data becomes complex [6] and thus has limitations in the ability to extract meaningful subgroups from the complex and multidimensional neuropsychological data.

**Fig 1:**
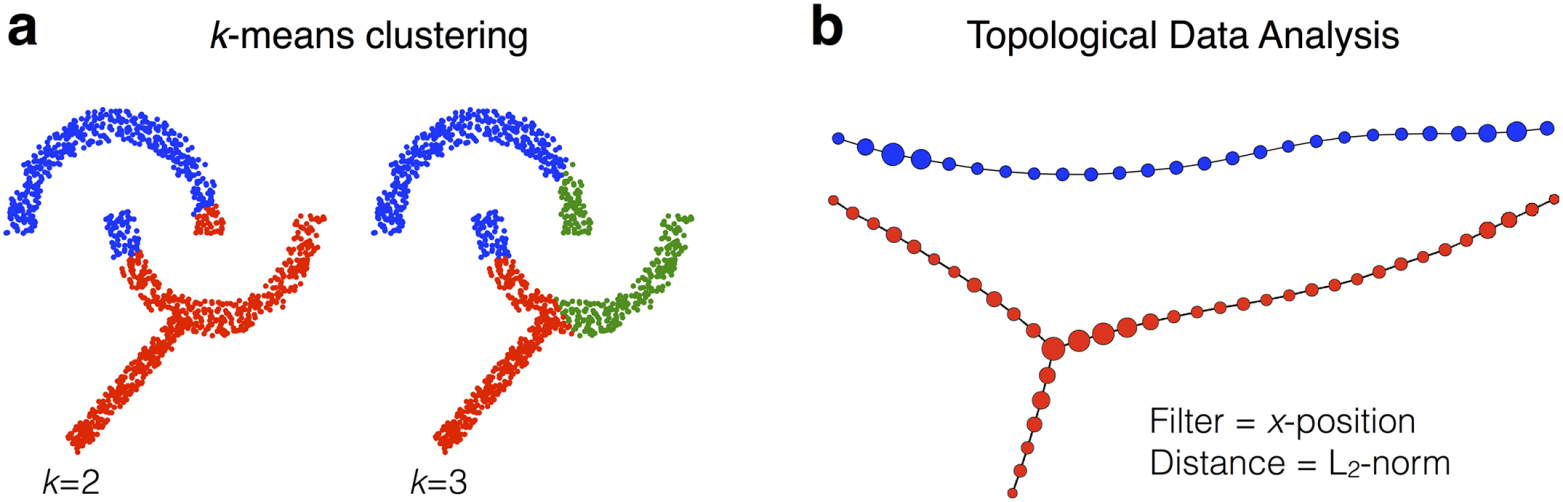
*K*-means clustering analysis with two (*k* = 2) and three (*k* = 3) centroids (a) and topological data analysis as an example of shape extraction (b). The classical *k*-means clustering fails to detect subgroups with complex shape of data.

Because of the above-mentioned limitations of the traditional clustering algorithm, Topological Data Analysis (TDA) is receiving increasing attention for its ability to define the shape of the data and produce outcomes in the form of an easily recognizable graph, as graphically illustrated in Fig 1. TDA, or “partial clustering”, which has been widely used for obtaining new insights from the heterogeneous high dimensional behavioral, clinical and biological datasets [7–12]. As an unsupervised machine learning approach, TDA has the attractive features of being data driven, without first having to formulate a pre-defined hypothesis. TDA can more finely stratify patients than standard clustering methods and can identify new as well as interesting patient sub-groups [10].

With regard to input features, several studies have attempted to identify ADHD subgroups by clustering them according to symptom severity [13, 14], reading disabilities [15], personality traits [16], anxiety/mood symptoms [17], and neuropsychological [18] and neuroimaging data [19] using data-driven machine learning algorithms. In our previous study, we have attempted to use a functional connectome-based topological data analysis (TDA) method to unveil hidden subgroups of ADHD, but failed to detect ADHD subgroups as a branch in the topology from neuroimaging data [19]. Instead, subgroup-like patterns have been observed when we mapped IQ and ADHD symptom severity on the resulting topology. In addition, previous studies demonstrated that general cognitive ability (intelligence quotient; IQ) and the severity of ADHD symptoms are two of the most important factors related to the prognosis of ADHD [20]. Specifically, individuals with high levels of ADHD symptoms are at an increased risk of continuing problems associated with ADHD during adolescence [21, 22]. It is also reported that IQ, but no other cognitive measures, affects ADHD outcome and that lower IQ is associated with comorbid conduct disorder, poor response to stimulant treatment, and poor prognosis [23, 24]. In fact, commonly measured basic neurocognitive and clinical characteristics, such as IQ and symptom severity, have their own advantages in that they can be more easily translated into clinical application [23] than that of neuroimaging data. Besides, these measures are best represented as dimensions and have well-theorized relationships to biological systems from previous studies [23, 25]. Therefore, IQ and symptom severity measures were selected to identify hidden subgroups using TDA in this study.

After identifying subgroups within large ADHD datasets, the investigation of the neurobiological validity of the newly defined subgroups is another important issue. In prior studies, neuroimaging methods, such as resting state functional magnetic resonance imaging (rs-fMRI) connectivity strength, were used to investigate the neurobiological substrates of human characteristics, such as personality types [4, 26]. A benefit of rs-fMRI is that it is more developmentally and contextually variable than brain structural measures, making it a highly attractive place to start working towards the goal of identifying subgroups of individuals with ADHD based on similar brain processes [27]. The presence of the subgroups that correspond to well-known brain functional systems identified by MRI implies that those subgroups may model some features of brain organization, and the absence of such subgroups may suggest that a subgroup may not be well-defined biologically [28].

On this background, the two main objectives of this study were (1) to determine whether we can identify new clinical phenotypes based on symptom severity and IQ measurements in children and adolescents with ADHD, and (2) to investigate whether neuroimaging findings validate the identified phenotypes. In this study, we used two brain-based measures to investigate the characteristics of the brains in each subgroup identified by TDA: 1) centrality measures, and 2) modularity analysis, exploring resting-state functional connectivity in ADHD in five pre-defined neural networks, searching for the neurobiological significance of the subgroups identified by TDA.

## Materials and methods

### Principal and validation datasets

We obtained the preprocessed rs-fMRI data from the Neuro Bureau ADHD-200 Preprocessed repository [29]. In the ADHD-200 dataset, the largest dataset from Peking University (PKU), containing both typically developing controls (TDCs) and children with ADHD, was chosen for this study. Among 176 male subjects, we selected 62 TDCs and 67 children with ADHD, choosing only those with complete fMRI, IQ, and clinical datasets without missing variables. The principal dataset contained the neuroimaging network data as well as the clinical measures, such as ADHD symptom severity and IQ scores. For the validation of the proposed topology-based methods, we chose the second largest dataset. From the 173 male subjects included in the New York University (NYU) dataset, we selected 43 TDCs and 91 children with ADHD, choosing only those with complete Q and symptom severity of ADHD datasets without missing variables. To control the potential confounding effects of sex and IQ, we excluded girls and subject***s*** with IQs lower than 80. The ADHD Rating Scale IV [30] and the Conners’ Parent Rating Scale-Revised [31] were used to assess symptom severity in the principal and validation datasets, respectively. The Wechsler Intelligence Scale for Chinese Children-Revised [32] and Wechsler Abbreviated Scale of Intelligence [33] were used to assess intelligence ability in the principal and validation datasets, respectively. Participant demographic and clinical information are shown in Table 1 and S1 Table online. All experimental protocols were in compliance with the policies of site-specific institutional review boards (IRBs). The ADHD-200 datasets are anonymized, with no protected health information included. Therefore, this study met the requirements for exemption from our local IRB review.

**Table 1:** Demographic and clinical characteristics of the principal dataset.

Variable	TDC, *a*	mADHD, *b*	sADHD, *c*	*F*_2,42_	*P*-value	*post-hoc*
	Mean ± SD	Mean ± SD	Mean ± SD			
Age	12.3 ± 1.6	13.1 ± 1.4	11.9 ± 1.6	2.2	0.129	
Intelligence quotient (IQ)						
Full-scale IQ	123.0 ± 15.0	102.9 ± 8.0	104.9 ± 13.9	11.4	<0.001	*a*>*b*, *a*>*c*
Verbal IQ	125.8 ± 14.3	107.9 ± 10.9	110.7 ± 20.1	5.7	0.006	*a*>*b*, *a*>*c*
Performance IQ	114.8 ± 15.1	96.7 ± 10.2	96.8 ± 12.4	10.1	<0.001	*a*>*b*, *a*>*c*
Symptom severity						
ADHD index	22.4 ± 2.9	44.1 ± 3.0	63.3 ± 2.8	753.5	<0.001	*a*<*b*, *a*<*c*, *b*<*c*
Inattentive	11.6 ± 1.9	26.1 ± 2.7	32.1 ± 2.2	324.2	<0.001	*a*<*b*, *a*<*c*, *b*<*c*
Hyper/Impulsivity	10.8 ± 2.1	18.1 ± 2.5	31.2 ± 2.9	253.5	<0.001	*a*<*b*, *a*<*c*, *b*<*c*
DSM-IV subtype, N (%)						
Combined		4 (27%)	12 (80%)			
Inattentive		11 (73%)	3 (20%)			
Hyperactive		0 (0%)	0 (0%)			

Abbreviation: ADHD, attention deficit hyperactivity disorder; DSM-IV, Diagnostic and Statistical manual of Mental Disorders, Fourth Edition; TDC, typically developing control; SD, standard deviation.

### Topological data analysis (TDA)

**Fig 2:**
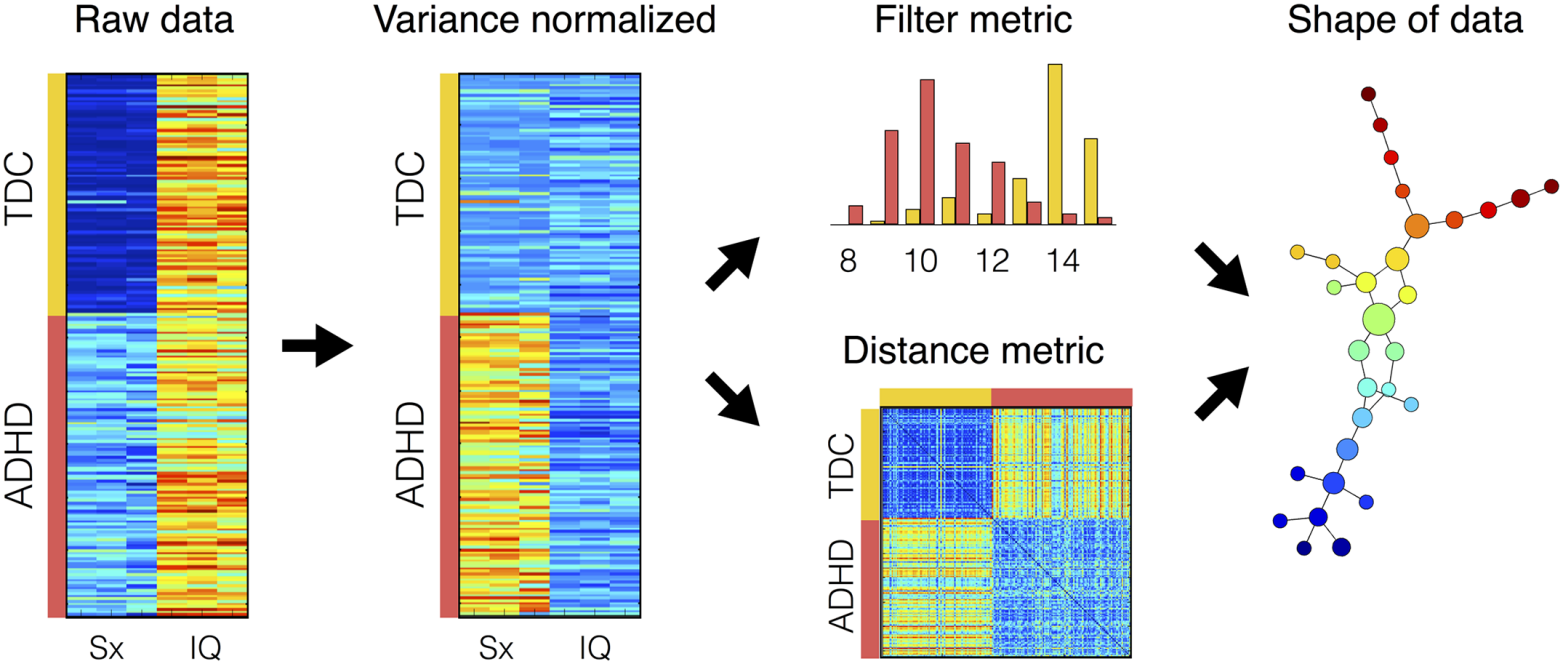
Preprocesses and analysis steps of topological data analysis. Abbreviations: ADHD, attention-deficit/hyperactivity disorder; TDC, typically developing controls; Sx, symptom severity scores; IQ, intelligence quotient.

We performed TDA to identify hidden subgroups of ADHD. We prepared a dataset as a matrix form using three clinical variables for symptom severity and three variables for intelligence. Therefore, the matrix (*M*_*ij*_) indicates a point of data for a subject *i* and a variable *j*, where characteristic variables contained three symptom variables, such as ADHD index, inattentive score and hyperactive/impulsivity score, and three intelligence scores, such as full-scale IQ, verbal IQ and performance IQ. Fig 2 shows the preprocessing and analysis steps of TDA. At first, the means and standard deviations of the TDCs were computed as following:

$$\mu_{j}^{\text{TDC}}=\frac{1}{N_{\text{TDC}}}\sum_{i\in X_{\text{TDC}}}M_{ij}\;\text{and}\;\sigma_{j}^{\text{TDC}}=\sqrt{\frac{1}{N_{\text{TDC}}}\left(M_{ij}-\mu_{j}^{\text{TDC}}\right)}$$ (1)

where *N*_TDC_ is the number of subjects in TDCs; *X*_TDC_ is a subset of subjects in TDC. Finally, the variance normalized data matrix (${\hat{M}}_{ij}$) was computed as

$${\hat{M}}_{ij}=\frac{M_{ij}-\mu_{j}^{\text{TDC}}}{\sigma_{j}^{\text{TDC}}}$$ (2)

Now, the distance metrics between *α*- and *β*-th subjects were computed by Euclidean distance as following:

$$D_{\alpha \beta }=\|{\hat{M}}_{\alpha \circ }-{{\hat{M}}_{\beta \circ }\|}_{2}=\overset{6}{\underset{k=1}{\sum }}{\left({\hat{M}}_{\alpha k}-{\hat{M}}_{\beta k}\right)}^{2}$$ (3)

Then, we applied *L*-infinity centrality to compute filter metric as following:

$$f_{\alpha }=\underset{\beta \in X}{\text{max}}\left(D_{\alpha \beta }\right).$$ (4)

This computes the maximal distance to any other data points in the dataset *X* for every subject’ data point. Finally, *Mapper*, a tool for TDA, was used to find hidden subgroups in the patient-patient networks with the distance and filter metrics as input variables. After extracting the shape of the data, we mapped variables of interest onto the resulting topology, or patient-patient network, to unveil hidden neuropsychological characteristics of the output topology.

### Functional network matrix

The datasets that we downloaded from the Neuro Bureau ADHD-200 repository were the filtered time courses files, ADHD 200_AAL_TCs_filtfix.tar.gz. A detailed description of the preprocessing steps can be found elsewhere [19, 29]. In brief, slice timing correction, coregistration, normalization, smoothing, regressions to remove physiological noises and head motion artifacts, and band-pass (0.009–0.08 Hz) filtering were applied. Using the filtered time-course data, we scrubbed time points for huge head motions by removing the fMRI scans using a framewise displacement > 1 mm [34]. Then, the functional network of each subject (*R*_*ij*_) was computed by Pearson’s correlation coefficients of the scrubbed time-courses between the *i*-th and *j*-th regions of interest. These functional connectivity matrices were used for further investigation of functional network properties.

### Graph theoretical analysis

For the modularity optimization analysis and computation of centrality measures, we only used positive edge weights [26, 35]. The degree centrality of a node was computed by summation of all connection weights from its neighboring nodes [36]. The betweenness centrality and PageRank of a node were also computed following the definition described in elsewhere [36, 37]. The betweenness centrality of a node captures the global structure of the network. The PageRank evaluates the importance of nodes in network systems [38]. We set *α* to 0.85 for the evaluation of the PageRank.

To evaluate modular organization of each group, the group representing connection matrices were computed by averaging the functional network within each group. There are numerous approaches to compute the modular architectures in a network [39–42]. Among them, we adopted the *Louvain* community detection method that uses a heuristic algorithm to compute the modularity.

$$Q=\frac{1}{2m}\sum_{i,j}\left(A_{ij}-\frac{s_{i}s_{j}}{2m}\right)\delta \left(C_{i},C_{j}\right)$$ (5)

where *A*_*ij*_ is the connection weight of the edge between *i* and *j*; $m=\frac{1}{2}\sum_{ij}A_{ij}$; *s*_*i*_ = ∑_*j*_ *A*_*ij*_ is the degree centrality; *C*_*i*_ is the community structure to which node *i* is assigned; the delta function *δ*(*x*, *y*) is 1 if *x* = *y* and 0 otherwise. The heuristic modularity optimization algorithm produced slightly variable modular partitions and modularity (*Q*) run by run. Therefore, we performed 1000 independent optimization processes and found the best module partition that had the highest average value of normalized mutual information over all other optimization results [26].

### Statistical analysis

The statistical comparisons of demographic variables, symptom severity, and IQ scores among subgroups identified by TDA were conducted by analysis of variance (ANOVA) methods. In addition, group level differences of graph theoretical measures and the intra- and inter-modules functional connectivity density (FCD) were investigated by analysis of covariance (ANCOVA). In addition, the statistically meaningful FCDs and centrality measures among the three groups were identified using a statistical significance of corrected *P* < 0.05, correcting for multiple comparisons using the Benjamini-Hochberg procedure [43]. Further investigations of the meaningful FCDs and centrality measures (*P* < 0.05, Bonferroni-corrected) were conducted using a *post-hoc* analysis to identify differences between any combinations of the groups.

## Results

### Participant characteristics

In the principal and validation datasets, no significant differences were found in age and handedness between the ADHD and TDC groups (see S1 Table online). However, significantly higher IQ scores were observed for both datasets (*P* < 0.05) in TDC subjects compared to children with ADHD.

### Topological data analysis

**Fig 3:**
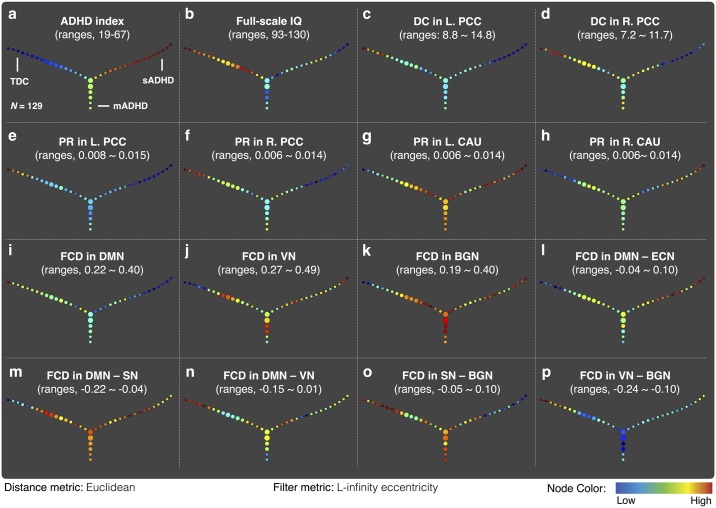
Output of topological data analysis of the principal dataset. The patient-patient network discriminates children with ADHD into two distinct groups in terms of symptom severity. The average values of various clinical and neuroimaging variables within each node were plotted. Abbreviations: ADHD, attention-deficit/hyperactivity disorder; BGN, basal ganglia network; CAU, caudate; DC, degree centrality; DMN, default mode network; FCD, functional connectivity density; IQ, intelligence quotient; L, left; PCC, posterior cingulate cortex; R, right; PR, PageRank; SN, salience network; VN, visual network.

Fig 3 presents the results of the TDA (or patient-patient network), showing a three branch-shaped graph for group separation in terms of the distance between the participants. TDA identified three subgroups of children: one TDC and two ADHD subgroups. The average values of the ADHD index and full-scale IQ within each node of the output topology are shown in Fig 3a and 3b. According to the variable mapping onto the patient-patient network, one branch in the patient-patient network of ADHD had milder inattentive and impulsivity/hyperactivity symptoms, labelled as mild ADHD (mADHD), and the other branch had more severe symptoms, labelled as severe ADHD (sADHD). Furthermore, for the quantitative analysis of the intelligence and ADHD symptoms among the three branches, we selected the 15 children lying on the most extreme peripheral position of each branch in the patient-patient networks. The demographic and clinical profiles for each subgroup are described in Table 1. Specifically, symptom severity of mADHD subgroup was significantly lower than that of sADHD subgroup (*P* < 0.05), whereas no significant differences in intelligence scores were observed between two ADHD subgroups (*P* > 0.05). The validation analysis confirmed that similar patterns in the patient-patient network were observed in the validation dataset. The shape of the data in the intelligence and symptom severity space obtained from the validation dataset was the same as the shape of the principal dataset, a three branch-shaped graph including severe and mild symptom ADHD subgroups (see S1 Fig online).

### Centrality measures among the three subgroups

The ANOVA test showed that the degree centrality and PageRank in the bilateral posterior cingulate cortex (PCC) were significantly different among the three groups (Corrected *P* < 0.05). The PageRank in the bilateral caudate was significantly different among the three groups (Corrected *P* < 0.05). The statistical comparisons of all nodal areas are summarized in supplementary materials (see S2 and S4 Tables online). Specifically, the degree centrality and PageRank in the bilateral PCC was significantly decreased in the sADHD group relative to that of the mADHD and TDC groups (Bonferroni-corrected *P* < 0.05). Interestingly, the PageRank in the bilateral caudate was significantly increased in both the mADHD and sADHD groups relative to the TDC group (Bonferroni-corrected *P* < 0.05). There were no significant differences in the betweenness centrality among three subgroups (S3 Table online). Furthermore, we mapped the degree centrality in the bilateral PCC (Fig 3c and 3d) and the PageRank in the bilateral PCC (Fig 3e and 3f) and bilateral caudate (Fig 3g and 3h) onto the patient-patient network to visualize the neural characteristics of each subgroup.

### Modularity optimizations and functional connectivity density (FCD)

**Fig 4:**
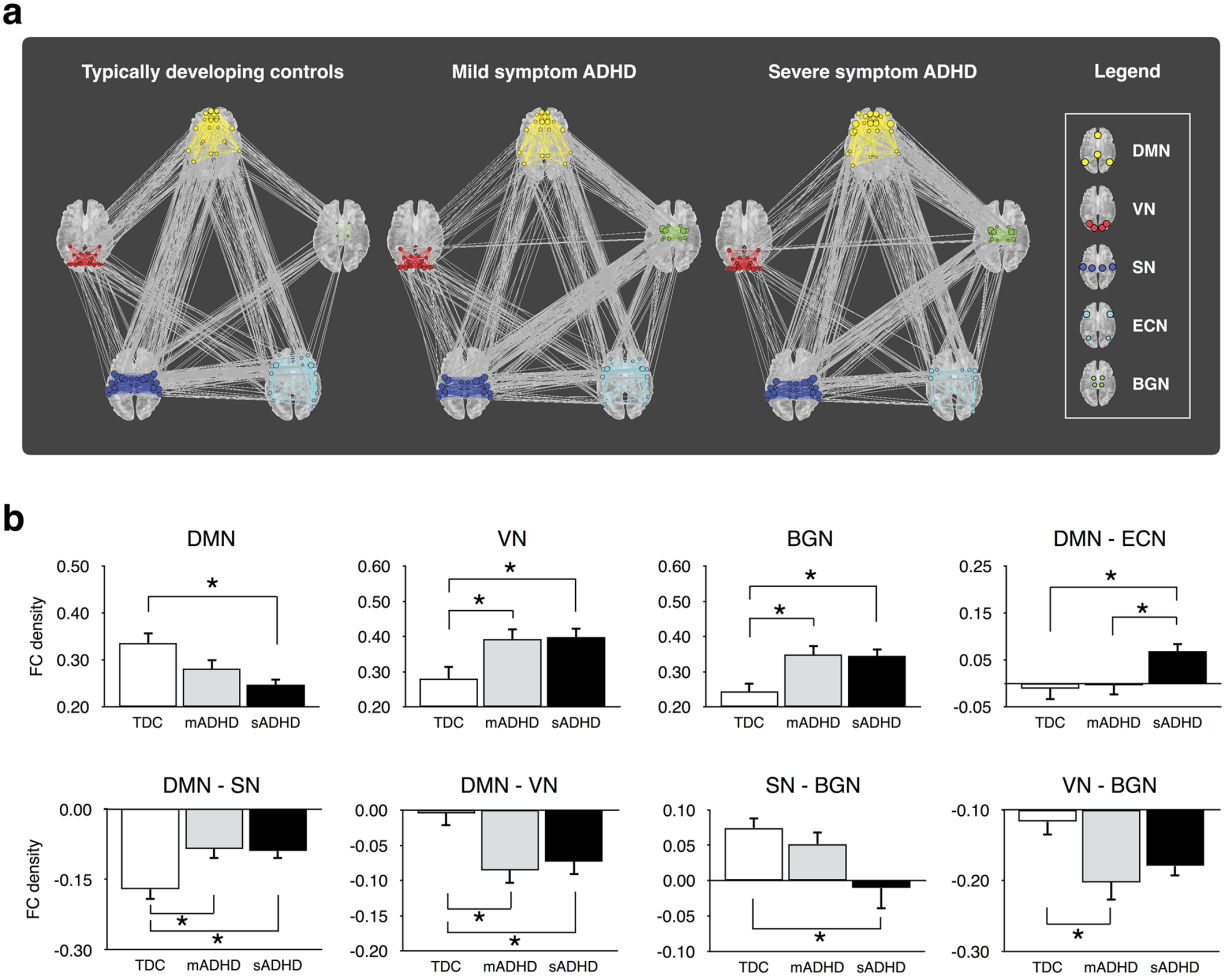
Functional network modular organizations for typically developing controls (TDC), children with mild symptom ADHD (mADHD), and children with severe symptom ADHD (sADHD) (a), and statistical comparisons of the functional connectivity density among the three subgroups (b). Modular partitions were obtained from the best community structure of the mADHD group and significant intra- and inter-module connectivities were plotted as bar graphs. *P < 0.05 indicates a statistical significance from the post-hoc analysis after Bonferroni correction. Abbreviations: ADHD, attention-deficit/hyperactivity disorder; BGN, basal ganglia network; DMN, default mode network; ECN, executive control network; SN, salience network; VN, visual network.

Fig 4a shows the patterns of the functional connectivity and modular organization for the three subgroups of ADHD. In brief, the default mode network (DMN), visual network (VN), salience network (SN), executive control network (ECN), and basal ganglia network (BGN) were identified in all subgroups, though each group had slightly different community membership. These memberships are described in S5 Table online. The functional communities obtained from the mADHD group were selected for statistical comparisons of FCD among the three groups. As shown in Table 2, the ANOVA test revealed significant group differences in the intra-module FCDs in the DMN, VN, and BGN (Corrected *P* < 0.05). Meanwhile, the inter-module FCDs in the DMN—ECN, DMN—SN, DMN—VN, SN—BGN, and VN—BGN were also significantly different among the three subgroups (Corrected *P* < 0.05).

**Table 2:** Analysis of covariance of the functional connectivity density (FCD) among three groups. The statistical significance of the intra-module and inter-module FCD were summarized.

Functional module	TDC	mADHD	sADHD	Analysis of Variance	
	Mean ± SD	Mean ± SD	Mean ± SD	*F*_2,42_	Corrected *P*^a^
Intra-module FCD					
Default Mode Network (DMN)	0.34 ± 0.09	0.28 ± 0.08	0.25 ± 0.05	5.54	0.03
Executive Control Network (ECN)	0.28 ± 0.06	0.28 ± 0.06	0.29 ± 0.09	0.02	0.98
Salience Network (SN)	0.34 ± 0.07	0.32 ± 0.06	0.36 ± 0.08	1.21	0.45
Visual Network (VN)	0.28 ± 0.14	0.39 ± 0.12	0.40 ± 0.11	4.51	0.04
Basal Ganglia Network (BGN)	0.24 ± 0.10	0.35 ± 0.10	0.34 ± 0.08	5.98	0.03
Inter-module FCD					
DMN—ECN	-0.01 ± 0.09	0.00 ± 0.08	0.07 ± 0.07	4.44	0.04
DMN—SN	-0.17 ± 0.08	-0.09 ± 0.08	-0.09 ± 0.07	5.87	0.03
DMN—VN	0.00 ± 0.07	-0.09 ± 0.07	-0.07 ± 0.07	5.57	0.03
DMN—BGN	0.02 ± 0.06	0.06 ± 0.05	0.07 ± 0.05	2.74	0.13
ECN—SN	0.00 ± 0.07	-0.02 ± 0.08	-0.03 ± 0.07	0.72	0.57
ECN—VN	-0.08 ± 0.05	-0.06 ± 0.08	-0.10 ± 0.10	1.14	0.45
ECN—BGN	-0.01 ± 0.07	-0.02 ± 0.06	0.01 ± 0.07	0.74	0.57
SN—VN	-0.11 ± 0.09	-0.08 ± 0.10	-0.09 ± 0.08	0.52	0.64
SN—BGN	0.07 ± 0.06	0.05 ± 0.07	-0.01 ± 0.12	4.08	0.05
VN—BGN	-0.12 ± 0.08	-0.20 ± 0.10	-0.18 ± 0.06	4.56	0.04

^*a*^Corrected *P* was obtained by Benjamini-Hochberg procedure to correct multiple comparisons.

Abbreviation: ADHD, attention deficit hyperactivity disorder; mADHD, children with mild symptom ADHD; sADHD, children with severe symptom ADHD; SD, standard deviation.

Fig 4b shows the significant intra- and inter-module FCDs among the three groups and presents the significance from the *post-hoc* analyses. Relative to the TDCs, the intra-module FCDs within the VN and BGN, and inter-module FCD in the DMN—SN were significantly increased in the mADHD as well as the sADHD groups (Corrected *P* < 0.05). Meanwhile, the intra-module FCD within the DMN was significantly decreased in the sADHD group compared to the TDC groups (Corrected *P* < 0.05). In contrast, the inter-module FCD in the DMN—VN was significantly decreased in the mADHD and sADHD groups relative to the TDCs (Corrected *P <* 0.05). In addition, the inter-module FCD in the SN—BGN was significantly decreased in the sADHD relative to the TDC group (Corrected *P* < 0.05), and the inter-module FCD in the VN—BGN was significantly decreased in the mADHD relative to the TDC group (Corrected *P* < 0.05). Finally, the inter-module FCD of the DMN—ECN in the sADHD group was significantly increased relative to that of the TDC and mADHD groups (Corrected *P* < 0.05). These significant FCD values were mapped onto the patient-patient network, as shown in Fig 3i–3p. Visualization of the variables on the topology provided a complementary information to the F-test among the three groups.

## Discussion

In the present study, we demonstrated that the use of common clinical phenotypes (symptom severity and IQ) and an innovative unsupervised data-driven machine learning algorithm such as TDA is an informative approach for understanding the heterogeneity of ADHD. We also examined whole-brain intrinsic functional connectivity and identified the specific brain network that shows significant differences between the mADHD group and the sADHD or the TDC groups. This result suggests that the identification of the mADHD and sADHD subgroups may be biologically meaningful when the functional connectome is examined using graph theoretical approaches.

### Subgroup identification of ADHD by TDA

Our results showed that TDA produced an easily recognizable branch-shaped graph with three progressive arms and that individuals with ADHD in the same arm shared similar characteristics to form specific subgroups of the disorder. Similar results were also replicated in the independent validation dataset. The most compelling finding of this study is the identification of two unique phenotypes (mADHD and sADHD), which overlaps with current ADHD categories identified in the Diagnostic and Statistical Manual of Mental Disorders, Fifth Edition (DSM-5) [44], *i*.*e*., combined, inattentive, and hyperactive/impulsive presentation. As shown in Table 1 (see also S2 Fig online), most of the sADHD (80%) and mADHD (73%) overlapped with the combined subtype and the inattentive subtype, respectively. Given that the wording ‘subtype’ that was used in the DSM-IV to categorize the children with ADHD has been changed as ‘presentation’ in the DSM-5 [44], the mADHD and sADHD identified by TDA might be called as predominately inattentive presentation and predominantly combined presentation, respectively. In fact, the combined subtype was clinically more severe than the inattentive subtype (see S6 Table online). However, these two subtypes showed no significant differences in the functional network measures (see S7–S10 Tables online). Taken together, although mADHD and sADHD showed overlaps in the DSM-IV subgroups or DSM-5 presentation, they are ‘novel subgroups’ regards to showing significant differences in the functional network measures as well as symptom severity.

In our previous study, we have failed to detect subgroups of ADHD using the functional connectome data as the input features [19]. Healthy state modeling, which introduced in the first TDA paper that identified a subgroup of breast cancers [11], was used to extract disease components from the functional imaging data. Then, the filter metric was computed as the size of disease component. The filter metric might have captured the overall characteristic of ADHD, but failed to capture additional information to classify subgroups of ADHD. However, the current study used easily obtainable clinical measures, such as ADHD symptom severity and IQ scores, as the input features to the TDA. We used *L*-infinity centrality as the filter metric, which computes for every data point the maximal distance to any other data point in the set, and thus the extreme (highest or lowest) filter metric corresponds to flares (or branches) in the patient-patient network of the TDA output. Therefore, the current approach has an advantage in being readily translated into clinical practice. Notably, these two subgroups displayed both similar and distinct functional connectivity findings in whole-brain and subnetwork connectivity, suggesting the biological validity of our subgroups. It is worth noting that the advantage of TDA is the ease with which it can be used to visualize similarities and differences between the mADHD and sADHD groups by mapping variables of interest, as shown in Fig 3.

### Functional network modular organization

In the analysis of functional network modular organization for the mADHD, sADHD, and TDC groups, we observed altered functional connectivity within several large-scale brain networks in children with ADHD compared with TDCs. Specifically, relative to the TDC group, the sADHD group displayed increased intra-module connectivity in the VN and BGN, and inter-module connectivity in the DMN—ECN, DMN—SN, DMN—VN, but decreased intra-module connectivity in the DMN and inter-module connectivity in the SN—BGN. Our results also revealed that the mADHD group generally showed similar patterns of modular architectures to that of the sADHD group. For example, intra-module connectivity in the VN and BGN, and inter-module connectivity in the DMN—SN and DMN—VN were not significantly different between the mADHD and sADHD groups, but the values were significantly different between mADHD and TDC groups. Taken together, our results suggest that both intra-module and inter-module functional connectivity are critical components of the underlying neurobiology of ADHD. In general, abnormal intra- and inter-module connectivity may limit dynamic interactions among networks, which are necessary for successful processing of complex stimuli in the real world [45].

Regarding the hypoconnectivity within the DMN and the decreased degree centrality and PageRank in the bilateral PCC regions, our results support the idea that ADHD might be considered as a default network disorder [46–50]. The altered inter-module connectivity between the DMN and the task-positive regions found in our ADHD group may be associated with a dysfunction in the switching of the brain state from a resting to functioning state [50], thus producing periodic lapses in on-task performance, which is a hallmark of ADHD [51].

Focusing on the subcortical system of ADHD, previous study associated the malfunction of the cognitive control in ADHD with abnormality in the striatal circuits [52]. The mADHD and sADHD showed an increase in PageRank in the bilateral caudate (S4 Table online) and FCD within the BGN (Fig 2b), which includes the striatum and thalamus, relative to the TDC group. The striatum has multiple roles relevant to ADHD such as executive function [53] and reward processing [54]. Considering that the PageRank measures an importance of a node in the network system [37], the increase in PageRank relative to the normal condition might be interpreted as information overload and might be associated with dysfunction in the striatal circuits.

Instead of the traditional simple models of ADHD, mainly involving DMN—ECN FC or within-DMN FC, our findings with regard to the VN and SN are consistent with the recent neurobiological formulations of ADHD [49, 55–57], implicating atypical functional connectivity within and between networks affecting sensory and salience processing. This theory suggests that enhanced bottom-up processing of external stimuli, as well as top-down processing of executive control, can contribute to increased distractibility in ADHD patients [57, 58]. Specifically, the visual cortex interacts with the dorsal attention network [59, 60] to maintain attention and suppress attention to irrelevant stimuli [55, 61]. Therefore, the abnormal intra- and inter-module VN connectivity found in our mADHD and sADHD groups may affect poor inhibition of sensory perception in ADHD patients [62–66]. The salience network integrates external sensory stimuli with internal states, and it is critical for attention allocation to stimuli that are salient to the individual [67] and units conflict monitoring, interoceptive-autonomic, and reward processing centers [68]. In addition, diminished DMN connections with the VN and SN may suggest that there is reduced information transfer from the sensory or salience network to the DMN [69]. The role of BGN has been less studied compared to that of other networks, but it has been implicated in working memory, motivation and reward-related processing in prior studies [70]. Taken together, our results are consistent with the current multi-network models in ADHD, which suggest that many psychiatric conditions, including ADHD, are characterized by inappropriate engagement of the SN with the ECN, DMN, VN, and BGN [55, 68, 71].

### Neural characteristics of our mADHD and sADHD subgroups

Our study results revealed that aberrant connectivity in the DMN, VN, BGN, ECN, or SN mainly concerned our sADHD group. The mADHD group shared neural signatures with the TDC group as well as the sADHD group. First, the degree centrality and PageRank in the bilateral PCC and intra-module connectivity in the DMN of the mADHD group were found to be intermediate between the sADHD and TDC groups. In contrast, relative to the TDC group, inter-module hypoconnectivity in the DMN—VN, inter-module hyperconnectivity in the DMN—SN, and intra-module hyperconnectivity in the VN and BGN were observed in both the mADHD and sADHD groups. However, inter-module hyperconnectivity in the DMN—ECN was observed in the sADHD group, but not in the mADHD group compared to TDC group. These findings show that mADHD and sADHD are somewhat different in terms of their DMN—ECN connectivity, suggesting neurobiological differences between these two subgroups. The DMN and ECN are known as the task negative and task positive regions, respectively, indicating that they should be anti-correlated in the normal brain [72]. Therefore, ADHD subjects with mild symptoms might be different from those with severe symptoms and more similar to TDC in terms of their DMN—ECN network.

Despite the important findings, several limitations should be considered for the proper interpretation of our results. Our findings cannot be extended to the entire ADHD population because we only used samples from boys to avoid possible bias from sex differences [73]. Furthermore, in this analysis of archival data, we were limited by the content of the datasets available. In the ADHD-200 datasets, medication status was missing for a large number of subjects, including the doses and types of medications and whether the subjects were under the effects of the medication during the fMRI scan. A history of treatment with stimulant medication may be relevant in interpreting our results. In addition, significantly higher IQ scores in TDC subjects than children with ADHD were observed in the principal and validation datasets. As individual differences in intrinsic functional connectivity are systematically related to measures of IQ [74] and behavioral variability [75], it is possible that the identified differences may be due to IQ differences rather than differences in symptom severity. The selection of the 15 subjects at the extreme of each subgroup was somewhat arbitrary. However, this was a *post-hoc* comparison and selection of 15 subjects is enough to produce statistically significant results. Our findings are somewhat dissimilar to the results from prior studies reporting reduced connectivity between the DMN and other networks of brain, such as the ECN [46–49]. These discrepancies are explained by the developmental perspective that suggests that DMN connections known to increase with development are weaker in participants with ADHD, and conversely, that a DMN connection known to decrease with development is abnormally increased in the children with ADHD [47, 49]. Therefore, further work with larger samples, tighter age ranges, and more standardized protocols for diagnosis is needed and the effects of comorbidities should be considered in future studies.

### Conclusion

Despite the limitations mentioned above, we showed two notable findings in this study. First, TDA is potentially useful in defining less heterogeneous clinical subgroups in ADHD. Second, neuroimaging measurements are potential validators of phenotypic groups identified using behavioral data. Taken together, data-driven and biologically relevant disease phenotypes identified using TDA will give researchers a unique opportunity to identify objective and biologically relevant disease categories. The identification of subgroups with distinct patterns of clinical manifestation may lead to development of more effective and better-targeted intervention for individualized treatment and improved patient care.

## Supporting information

S1 Fig Output of topological data analysis (TDA) of the validation dataset. The patient-patient network discriminates children with ADHD into two distinct groups in terms of symptom severity. The average values of full-scale IQ and ADHD index within each node were plotted. Abbreviations: ADHD, attention-deficit/hyperactivity disorder; IQ, intelligence quotient; NYU, New York University.
(DOCX)

S2 Fig Mapping clinical subtypes on the patient-patient network produced by topological data analysis (TDA) of the principal dataset. The patient-patient network discriminates children with ADHD into two distinct groups regarding symptom severity. Most severe symptom ADHD (sADHD) were the clinically combined subtypes, whereas most mild symptom ADHD (mADHD) were the clinically inattentive subtype. Each node indicates a group of subjects with similar characteristics in dimensions of symptom severity and intelligence score. Node color indicates the ratio of subjects with a clinical subtype. Abbreviations: ADHD, attention-deficit/hyperactivity disorder; IQ, intelligence quotient; PKU, Peking University.
(DOCX)

S1 Table Demographic and clinical characteristics of the principal and validation datasets. (DOCX)

S2 Table Mean values of degree centrality for each mADHD and sADHD subgroup and its statistical comparison using analysis of variance. (DOCX)

S3 Table Mean values of betweenness centrality for each mADHD and sADHD subgroup and its statistical comparison using analysis of variance. (DOCX)

S4 Table Mean values of PageRank for each mADHD and sADHD subgroup and its statistical comparison using analysis of variance. (DOCX)

S5 Table Functional modular organization for each mADHD and sADHD subgroup. Modularity optimization analyses were conducted for the principal data set.
(DOCX)

S6 Table Demographic and clinical characteristics of the inattentive and combined subtypes. (DOCX)

S7 Table Two-sample t-test of the functional connectivity density (FCD) between the inattentive and combined subtypes. The statistical significance of the intra-module and inter-module FCD were summarized.
(DOCX)

S8 Table Mean values of degree centrality for each inattentive and combined subtype and its statistical comparison using analysis of variance. (DOCX)

S9 Table Mean values of betweenness centrality for each inattentive and combined subtype and its statistical comparison using analysis of variance. (DOCX)

S10 Table Mean values of PageRank for each inattentive and combined subtype and its statistical comparison using analysis of variance. (DOCX)
