# Supplementary material for: Novel subgroups of attention-deficit/hyperactivity disorder identified by topological data analysis and their functional network modular organizations
Source: PLoS One. 2017 Aug 22;12(8):e0182603. doi: 10.1371/journal.pone.0182603 (PMC5567504; doi:10.1371/journal.pone.0182603)
Supplement: S1 Fig — The patient-patient network discriminates children with ADHD into two distinct groups in terms of symptom severity. The average values of full-scale IQ and ADHD index within each node were plotted. Abbreviations: ADHD, attention-deficit/hyperactivity disorder; IQ, intelligence quotient; NYU, New York University. (DOCX) [file pone.0182603.s001.docx]

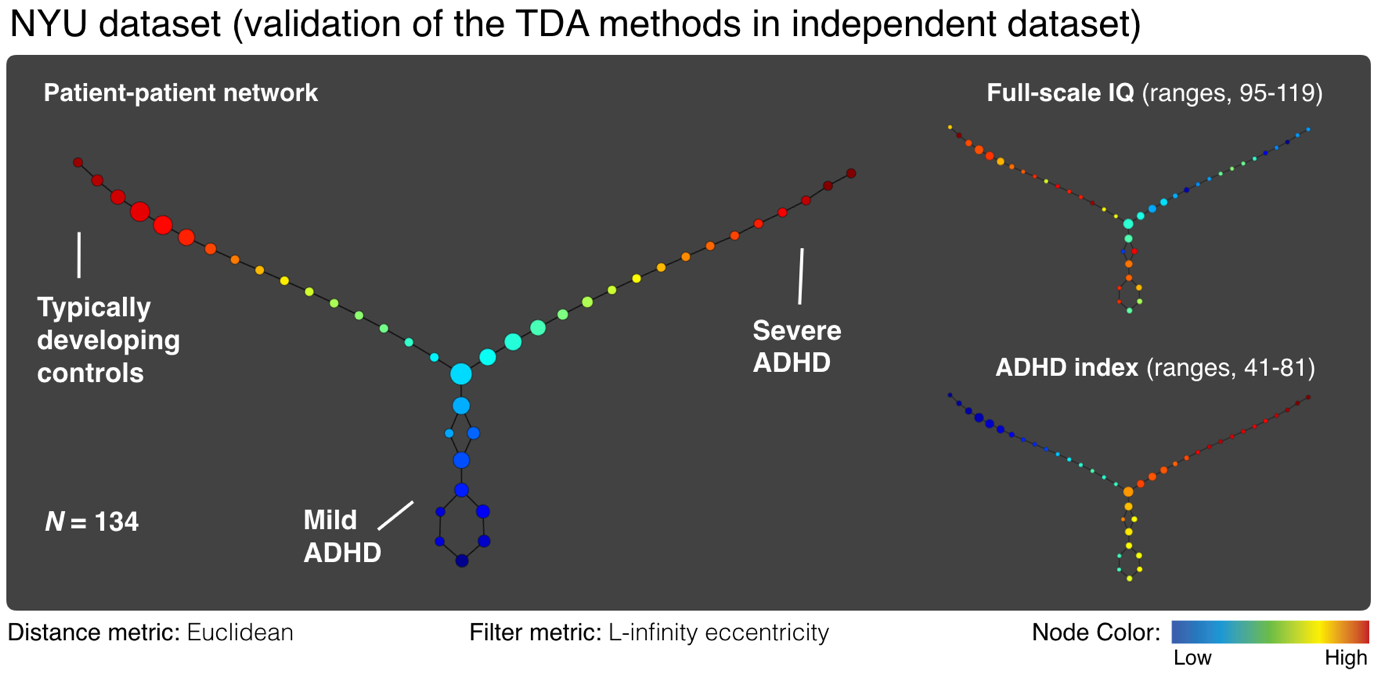


**S1 Fig**. **Output of topological data analysis (TDA) of the validation dataset**. The patient-patient network discriminates children with ADHD into two distinct groups in terms of symptom severity. The average values of full-scale IQ and ADHD index within each node were plotted.

Abbreviations: ADHD, attention-deficit/hyperactivity disorder; IQ, intelligence quotient; NYU, New York University.
