# Supplementary material for: Novel subgroups of attention-deficit/hyperactivity disorder identified by topological data analysis and their functional network modular organizations
Source: PLoS One. 2017 Aug 22;12(8):e0182603. doi: 10.1371/journal.pone.0182603 (PMC5567504; doi:10.1371/journal.pone.0182603)
Supplement: S2 Fig — The patient-patient network discriminates children with ADHD into two distinct groups regarding symptom severity. Most severe symptom ADHD (sADHD) were the clinically combined subtypes, whereas most mild symptom ADHD (mADHD) were the clinically inattentive subtype. Each node indicates a group of subjects with similar characteristics in dimensions of symptom severity and intelligence score. Node color indicates the ratio of subjects with a clinical subtype. Abbreviations: ADHD, attention-deficit/hyperactivity disorder; IQ, intelligence quotient; PKU, Peking University. (DOCX) [file pone.0182603.s002.docx]

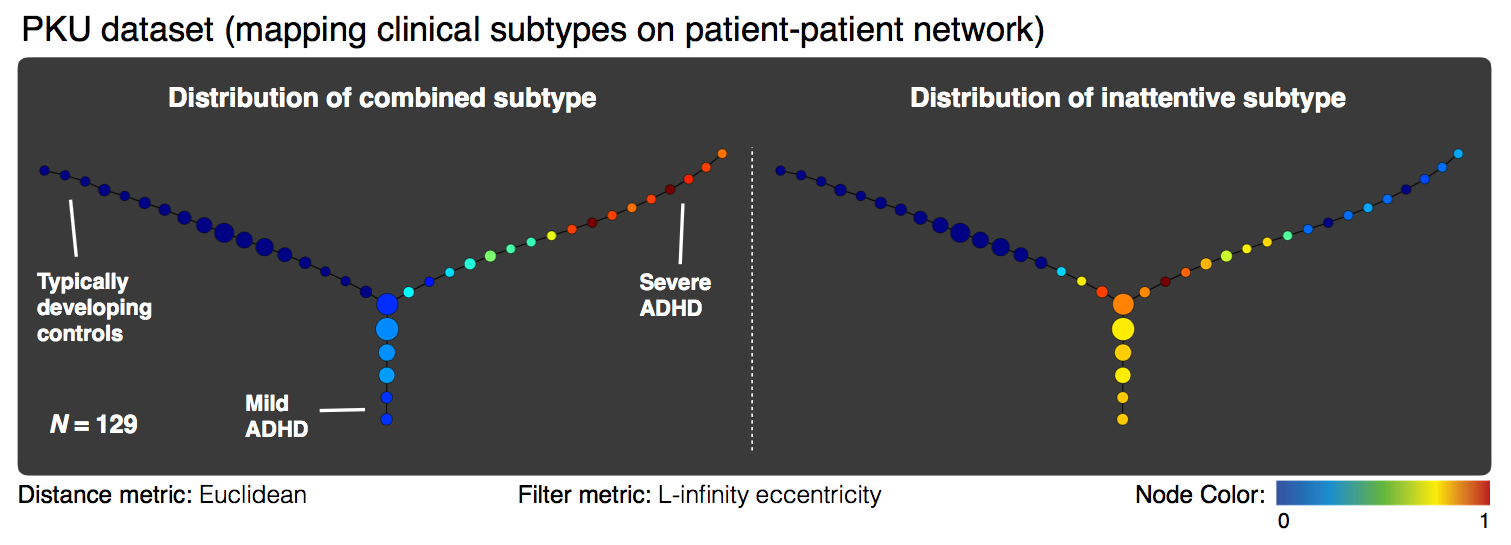


**S2 Fig**. **Mapping clinical subtypes on the patient-patient network produced by topological data analysis (TDA) of the principal dataset**. The patient-patient network discriminates children with ADHD into two distinct groups regarding symptom severity. Most severe symptom ADHD (sADHD) were the clinically combined subtypes, whereas most mild symptom ADHD (mADHD) were the clinically inattentive subtype. Each node indicates a group of subjects with similar characteristics in dimensions of symptom severity and intelligence score. Node color indicates the ratio of subjects with a clinical subtype.

Abbreviations: ADHD, attention-deficit/hyperactivity disorder; IQ, intelligence quotient; PKU, Peking University.
