# Supplementary material for: Novel subgroups of attention-deficit/hyperactivity disorder identified by topological data analysis and their functional network modular organizations
Source: PLoS One. 2017 Aug 22;12(8):e0182603. doi: 10.1371/journal.pone.0182603 (PMC5567504; doi:10.1371/journal.pone.0182603)
Supplement: S1 Table — (DOCX) [file pone.0182603.s003.docx]

**S1 Table. Demographic and clinical characteristics of the principal and validation datasets**

| Variable | Principal dataset from Peking University | | | |  | Validation dataset from New York University | | | |
| --- | --- | --- | --- | --- | --- | --- | --- | --- | --- |
|  | TDC | ADHD | *T* | *P*-value |  | TDC | ADHD | *T* | *P*-value |
|  | Mean ± SD | Mean ± SD |  |  |  | Mean ± SD | Mean ± SD |  |  |
| N | 62 | 67 |  |  |  | 43 | 91 |  |  |
| Age | 12.1 ± 1.6 | 12.4 ± 1.9 | -0.9 | 0.348 |  | 11.7 ± 3.2 | 11.4 ± 2.7 | 0.7 | 0.459 |
| Intelligence quotient (IQ) | | | | | | | | | |
| Full-scale IQ | 118.5 ± 13.8 | 105.3 ± 12.2 | 5.7 | <0.001 |  | 113.1 ± 14.3 | 107.0 ± 14.0 | 2.3 | 0.021 |
| Verbal IQ | 120.2 ± 13.4 | 110.7 ± 16.0 | 3.6 | <0.001 |  | 113.2 ± 13.5 | 107.8 ± 14.1 | 2.1 | 0.041 |
| Performance IQ | 112.5 ± 14.8 | 97.9 ± 13.2 | 5.9 | <0.001 |  | 110.1 ± 15.3 | 104.2 ± 14.0 | 2.2 | 0.031 |
| Symptom severity | | | | | | | | | |
| ADHD index | 28.5 ± 5.6 | 50.9 ± 8.9 | -17.0 | <0.001 |  | 45.2 ± 7.4 | 69.3 ± 7.1 | -18.1 | <0.001 |
| Inattentive | 15.5 ± 3.7 | 28.2 ± 3.7 | -19.6 | <0.001 |  | 45.0 ± 7.2 | 68.8 ± 8.2 | -16.4 | <0.001 |
| Hyper/impulsivity | 13.1 ± 3.4 | 22.8 ± 6.5 | -10.5 | <0.001 |  | 45.2 ± 4.6 | 67.2 ± 11.3 | -12.3 | <0.001 |
| Subtype, N(%) |  |  |  |  |  |  |  |  |  |
| Combined |  | 27 (40%) |  |  |  |  | 61 (67%) |  |  |
| Inattentive |  | 40 (59%) |  |  |  |  | 28 (31%) |  |  |
| Hyperactive |  | 0 (0%) |  |  |  |  | 2 (2%) |  |  |

Abbreviation: ADHD, attention deficit hyperactivity disorder; TDC, typically developing control; SD, standard deviation.
