# Supplementary material for: Novel subgroups of attention-deficit/hyperactivity disorder identified by topological data analysis and their functional network modular organizations
Source: PLoS One. 2017 Aug 22;12(8):e0182603. doi: 10.1371/journal.pone.0182603 (PMC5567504; doi:10.1371/journal.pone.0182603)
Supplement: S2 Table — (DOCX) [file pone.0182603.s004.docx]

**S2 Table**. Mean values of degree centrality for each mADHD and sADHD subgroup and its statistical comparison using analysis of variance

| Anatomical Region | TDC | mADHD | sADHD | Analysis of Variance | |
| --- | --- | --- | --- | --- | --- |
|  | Mean ± SD | Mean ± SD | Mean ± SD | *F*_2,42_ | Corrected *P^a^* |
| Precentral gyrus (L) | 10.6 ± 2.4 | 9.3 ± 2.9 | 9.6 ± 2.3 | 0.99 | 0.858 |
| Precentral gyrus (R) | 11.8 ± 2.6 | 11.4 ± 2.4 | 12.6 ± 3.2 | 0.84 | 0.858 |
| Superior frontal gyrus (L) | 11.6 ± 2.8 | 10.5 ± 1.6 | 10.4 ± 2.7 | 1.27 | 0.858 |
| Superior frontal gyrus (R) | 9.1 ± 1.8 | 9.6 ± 2.2 | 10.3 ± 2.6 | 0.98 | 0.858 |
| Orbitofrontal cortex (superior) (L) | 13.7 ± 2.5 | 11.6 ± 2.1 | 12.9 ± 3.1 | 2.52 | 0.753 |
| Orbitofrontal cortex (superior) (R) | 11.7 ± 3.4 | 10.9 ± 2.5 | 12.9 ± 3.2 | 1.68 | 0.775 |
| Dorsolateral PFC (L) | 9.9 ± 2.1 | 9.4 ± 2.8 | 10.5 ± 2.4 | 0.86 | 0.858 |
| Dorsolateral PFC (R) | 9.7 ± 1.6 | 9.4 ± 1.9 | 10.8 ± 2.7 | 1.89 | 0.753 |
| Orbitofrontal cortex (middle) (L) | 11.1 ± 3.2 | 10.8 ± 2.6 | 12.6 ± 2.7 | 1.79 | 0.753 |
| Orbitofrontal cortex (middle) (R) | 11.6 ± 3.8 | 10.8 ± 2.0 | 12.9 ± 3.2 | 1.81 | 0.753 |
| Inferior frontal gyrus (operculuar) (L) | 11.5 ± 2.4 | 12.6 ± 3.4 | 12.5 ± 2.3 | 0.66 | 0.868 |
| Inferior frontal gyrus (opercular) (R) | 12.5 ± 2.9 | 12.5 ± 2.9 | 12.6 ± 2.7 | 0.01 | 0.995 |
| Inferior frontal gyrus (triangular) (L) | 11.7 ± 3.6 | 11.3 ± 2.9 | 12.1 ± 2.3 | 0.31 | 0.960 |
| Inferior frontal gyrus (triangular) (R) | 12.1 ± 2.8 | 12.0 ± 2.5 | 13.6 ± 3.1 | 1.63 | 0.780 |
| Inferior frontal gyrus (orbitalis) (L) | 13.6 ± 4.1 | 12.7 ± 3.2 | 14.5 ± 2.8 | 1.03 | 0.858 |
| Inferior frontal gyrus (orbitalis) (R) | 14.5 ± 4.0 | 14.2 ± 2.7 | 15.9 ± 3.2 | 1.08 | 0.858 |
| Rolandic operculum (L) | 14.6 ± 2.8 | 15.7 ± 1.8 | 14.5 ± 2.9 | 1.05 | 0.858 |
| Rolandic operculum (R) | 14.6 ± 2.5 | 15.5 ± 2.6 | 14.7 ± 2.9 | 0.51 | 0.925 |
| Supplementary motor area (L) | 11.3 ± 2.5 | 11.3 ± 2.4 | 11.1 ± 2.2 | 0.05 | 0.985 |
| Supplementary motor area (R) | 10.4 ± 3.3 | 10.0 ± 2.3 | 11.5 ± 2.3 | 1.29 | 0.858 |
| Olfactory (L) | 11.8 ± 3.3 | 10.9 ± 3.0 | 11.2 ± 3.3 | 0.35 | 0.960 |
| Olfactory (R) | 9.9 ± 3.6 | 9.7 ± 2.1 | 11.0 ± 3.6 | 0.73 | 0.858 |
| Dorsomedial PFC (L) | 13.9 ± 2.3 | 11.9 ± 1.9 | 12.6 ± 2.9 | 2.59 | 0.753 |
| Dorsomedial PFC (R) | 12.7 ± 2.5 | 11.4 ± 1.7 | 12.6 ± 2.6 | 1.45 | 0.855 |
| Ventromedial PFC (L) | 15.4 ± 2.2 | 13.6 ± 1.6 | 14.4 ± 3.0 | 2.26 | 0.753 |
| Ventromedial PFC (R) | 15.0 ± 2.4 | 13.1 ± 2.5 | 14.6 ± 3.0 | 2.09 | 0.753 |
| Rectus gyrus (L) | 15.5 ± 2.4 | 13.5 ± 2.1 | 14.5 ± 3.2 | 2.11 | 0.753 |
| Rectus gyrus (R) | 14.3 ± 2.4 | 12.7 ± 2.4 | 14.0 ± 3.7 | 1.39 | 0.858 |
| Insula (L) | 15.1 ± 3.8 | 15.0 ± 2.0 | 14.7 ± 2.8 | 0.08 | 0.985 |
| Insula (R) | 16.9 ± 3.1 | 16.9 ± 2.4 | 16.5 ± 2.3 | 0.10 | 0.985 |
| Ventral ACC (L) | 13.3 ± 2.1 | 12.8 ± 2.5 | 13.8 ± 2.5 | 0.75 | 0.858 |
| Ventral ACC (R) | 12.0 ± 3.1 | 12.9 ± 2.5 | 14.4 ± 2.8 | 2.86 | 0.680 |
| Dorsal ACC (L) | 10.1 ± 2.6 | 9.6 ± 2.7 | 10.2 ± 2.3 | 0.21 | 0.960 |
| Dorsal ACC (R) | 11.0 ± 2.4 | 10.0 ± 2.6 | 10.8 ± 1.8 | 0.73 | 0.858 |
| Posterior cingulate cortex (L) | 13.1 ± 2.6 | 11.4 ± 1.9 | 9.4 ± 1.7 | 11.67 | <0.001 |
| Posterior cingulate cortex (R) | 10.8 ± 2.5 | 9.6 ± 1.7 | 7.7 ± 1.4 | 9.51 | <0.001 |
| Hippocampus (L) | 10.2 ± 3.8 | 10.5 ± 2.7 | 9.5 ± 3.3 | 0.31 | 0.960 |
| Hippocampus (R) | 9.0 ± 4.5 | 9.1 ± 2.8 | 8.4 ± 2.5 | 0.20 | 0.960 |
| Parahippocampal gyrus (L) | 11.4 ± 3.5 | 10.0 ± 2.9 | 10.9 ± 2.7 | 0.88 | 0.858 |
| Parahippocampal gyrus (R) | 11.3 ± 3.2 | 11.7 ± 3.3 | 11.3 ± 2.1 | 0.14 | 0.985 |
| Amygdala (L) | 13.5 ± 3.6 | 14.6 ± 3.0 | 13.3 ± 2.9 | 0.76 | 0.858 |
| Amygdala (R) | 14.9 ± 3.9 | 15.0 ± 3.2 | 13.9 ± 2.8 | 0.47 | 0.925 |
| Calcarine cortex (L) | 10.7 ± 1.7 | 10.6 ± 2.3 | 10.4 ± 2.3 | 0.06 | 0.985 |
| Calcarine cortex (R) | 10.3 ± 2.3 | 11.0 ± 2.2 | 11.3 ± 2.4 | 0.82 | 0.858 |
| Cuneus (L) | 11.0 ± 2.1 | 10.4 ± 2.0 | 10.9 ± 2.1 | 0.37 | 0.958 |
| Cuneus (R) | 10.3 ± 1.7 | 11.0 ± 2.5 | 11.1 ± 2.8 | 0.42 | 0.925 |
| Lingual gyrus (L) | 10.5 ± 2.2 | 12.2 ± 3.3 | 12.1 ± 2.7 | 1.91 | 0.753 |
| Lingual gyrus (R) | 11.0 ± 2.4 | 12.3 ± 3.0 | 11.9 ± 2.7 | 0.88 | 0.858 |
| Superior occipital gyrus (L) | 10.1 ± 1.9 | 11.6 ± 2.4 | 11.5 ± 2.2 | 2.05 | 0.753 |
| Superior occipital gyrus (R) | 9.6 ± 1.6 | 10.4 ± 2.1 | 9.8 ± 1.5 | 0.80 | 0.858 |
| Middle occipital gyrus (L) | 10.6 ± 1.9 | 11.5 ± 1.6 | 11.2 ± 1.9 | 0.98 | 0.858 |
| Middle occipital gyrus (R) | 10.4 ± 2.2 | 11.4 ± 1.7 | 10.6 ± 1.7 | 1.11 | 0.858 |
| Inferior occipital gyrus (L) | 10.7 ± 2.0 | 11.3 ± 2.7 | 10.6 ± 1.8 | 0.48 | 0.925 |
| Inferior occipital gyrus (R) | 9.6 ± 2.2 | 10.5 ± 2.5 | 10.1 ± 1.5 | 0.69 | 0.858 |
| Fusiform gyrus (L) | 12.0 ± 2.8 | 12.3 ± 3.7 | 11.6 ± 3.1 | 0.21 | 0.960 |
| Fusiform gyrus (R) | 10.5 ± 2.5 | 12.0 ± 3.0 | 11.0 ± 2.4 | 1.30 | 0.858 |
| Postcentral gyrus (L) | 12.2 ± 2.2 | 12.2 ± 2.2 | 11.5 ± 2.2 | 0.48 | 0.925 |
| Postcentral gyrus (R) | 12.4 ± 2.4 | 12.4 ± 2.5 | 12.9 ± 3.2 | 0.21 | 0.960 |
| Superior parietal lobule (L) | 8.8 ± 1.4 | 9.4 ± 1.4 | 8.8 ± 2.0 | 0.69 | 0.858 |
| Superior parietal lobule (R) | 10.0 ± 1.2 | 9.4 ± 1.5 | 9.5 ± 2.1 | 0.53 | 0.925 |
| Inferior parietal lobule (L) | 9.8 ± 2.1 | 10.0 ± 1.6 | 9.8 ± 1.4 | 0.03 | 0.985 |
| Inferior parietal lobule (R) | 10.4 ± 2.5 | 10.4 ± 1.8 | 10.0 ± 2.0 | 0.22 | 0.960 |
| Supramarginal gyrus (L) | 12.7 ± 3.2 | 12.3 ± 2.4 | 11.8 ± 2.4 | 0.47 | 0.925 |
| Supramarginal gyrus (R) | 12.3 ± 3.2 | 11.9 ± 2.2 | 11.8 ± 1.5 | 0.23 | 0.960 |
| Angular gyrus (L) | 12.8 ± 2.5 | 11.0 ± 1.8 | 10.4 ± 2.1 | 5.05 | 0.270 |
| Angular gyrus (R) | 10.9 ± 2.0 | 10.5 ± 1.9 | 10.0 ± 2.4 | 0.59 | 0.912 |
| Precuneus (L) | 10.2 ± 1.9 | 9.2 ± 1.7 | 8.4 ± 1.0 | 4.61 | 0.285 |
| Precuneus (R) | 9.5 ± 1.9 | 8.8 ± 1.7 | 8.6 ± 1.3 | 1.11 | 0.858 |
| Paracentral lobule (L) | 7.9 ± 1.6 | 8.4 ± 2.4 | 9.5 ± 2.7 | 1.80 | 0.753 |
| Paracentral lobule (R) | 8.0 ± 1.5 | 8.6 ± 2.5 | 9.6 ± 2.2 | 2.31 | 0.753 |
| Caudate (L) | 8.0 ± 2.4 | 10.0 ± 2.6 | 10.4 ± 2.1 | 4.34 | 0.285 |
| Caudate (R) | 7.6 ± 2.9 | 10.0 ± 2.4 | 10.6 ± 2.9 | 4.94 | 0.270 |
| Putamen (L) | 15.3 ± 3.3 | 15.2 ± 3.0 | 14.9 ± 2.8 | 0.07 | 0.985 |
| Putamen (R) | 16.3 ± 3.0 | 16.0 ± 2.8 | 15.6 ± 3.0 | 0.25 | 0.960 |
| Pallidum (L) | 12.8 ± 1.7 | 13.5 ± 2.7 | 13.5 ± 2.7 | 0.43 | 0.925 |
| Pallidum (R) | 14.9 ± 2.4 | 14.8 ± 3.2 | 14.4 ± 2.0 | 0.16 | 0.982 |
| Thalamus (L) | 8.5 ± 2.7 | 11.0 ± 2.9 | 10.6 ± 2.8 | 3.55 | 0.450 |
| Thalamus (R) | 9.7 ± 3.1 | 10.2 ± 2.4 | 10.9 ± 2.4 | 0.87 | 0.858 |
| Heschl's gyrus (L) | 13.8 ± 2.7 | 14.4 ± 3.7 | 14.5 ± 2.8 | 0.23 | 0.960 |
| Heschl's gyrus (R) | 13.9 ± 2.3 | 14.2 ± 4.0 | 14.1 ± 2.0 | 0.04 | 0.985 |
| Superior temporal gyrus (L) | 15.4 ± 2.3 | 16.5 ± 3.2 | 15.4 ± 2.5 | 0.86 | 0.858 |
| Superior temporal gyrus (R) | 15.1 ± 2.6 | 15.6 ± 3.8 | 14.8 ± 2.9 | 0.27 | 0.960 |
| Temporal pole (superior) (L) | 16.3 ± 3.6 | 16.1 ± 3.8 | 16.1 ± 2.6 | 0.03 | 0.985 |
| Temporal pole (superior) (R) | 16.6 ± 3.3 | 16.8 ± 3.5 | 16.5 ± 2.6 | 0.04 | 0.985 |
| Middle temporal gyrus (L) | 12.9 ± 1.8 | 13.5 ± 2.5 | 11.9 ± 3.0 | 1.50 | 0.846 |
| Middle temporal gyrus (R) | 12.6 ± 2.8 | 12.4 ± 2.3 | 10.8 ± 3.2 | 1.76 | 0.753 |
| Temporal pole (middle) (L) | 13.5 ± 3.2 | 13.7 ± 3.5 | 13.2 ± 2.7 | 0.11 | 0.985 |
| Temporal pole (middle) (R) | 13.9 ± 3.0 | 14.5 ± 2.5 | 12.1 ± 2.3 | 3.49 | 0.450 |
| Inferior temporal gyrus (L) | 11.8 ± 3.3 | 11.3 ± 2.0 | 10.5 ± 2.7 | 0.85 | 0.858 |
| Inferior temporal gyrus (R) | 10.4 ± 2.3 | 10.8 ± 2.0 | 11.2 ± 2.9 | 0.42 | 0.925 |

*^a^*Corrected *P* was obtained by Benjamini-Hochberg procedure to correct multiple comparisons.

Abbreviation: ACC, anterior cingulate cortex; ADHD, attention-deficit/hyperactivity disorder; L, left; mADHD, mild symptom ADHD; PFC, prefrontal cortex; R, right; sADHD, severe symptom ADHD; SD, standard deviation; TDC, typically developing controls.
