# Supplementary material for: Novel subgroups of attention-deficit/hyperactivity disorder identified by topological data analysis and their functional network modular organizations
Source: PLoS One. 2017 Aug 22;12(8):e0182603. doi: 10.1371/journal.pone.0182603 (PMC5567504; doi:10.1371/journal.pone.0182603)
Supplement: S3 Table — (DOCX) [file pone.0182603.s005.docx]

**S3 Table**. Mean values of betweenness centrality for each mADHD and sADHD subgroup and its statistical comparison using analysis of variance

| Anatomical Region | TDC | mADHD | sADHD | Analysis of Variance | |
| --- | --- | --- | --- | --- | --- |
|  | Mean ± SD | Mean ± SD | Mean ± SD | *F*_2,42_ | Corrected *P^a^* |
| Precentral gyrus (L) | 0.032 ± 0.041 | 0.055 ± 0.064 | 0.037 ± 0.046 | 0.85 | 0.841 |
| Precentral gyrus (R) | 0.028 ± 0.040 | 0.031 ± 0.032 | 0.023 ± 0.034 | 0.16 | 0.941 |
| Superior frontal gyrus (L) | 0.026 ± 0.029 | 0.048 ± 0.057 | 0.040 ± 0.048 | 0.90 | 0.841 |
| Superior frontal gyrus (R) | 0.027 ± 0.031 | 0.044 ± 0.033 | 0.059 ± 0.084 | 1.23 | 0.841 |
| Orbitofrontal cortex (superior) (L) | 0.021 ± 0.022 | 0.041 ± 0.055 | 0.019 ± 0.017 | 1.71 | 0.841 |
| Orbitofrontal cortex (superior) (R) | 0.046 ± 0.048 | 0.031 ± 0.038 | 0.036 ± 0.030 | 0.56 | 0.859 |
| Dorsolateral PFC (L) | 0.053 ± 0.075 | 0.050 ± 0.090 | 0.023 ± 0.023 | 0.86 | 0.841 |
| Dorsolateral PFC (R) | 0.050 ± 0.088 | 0.050 ± 0.088 | 0.027 ± 0.053 | 0.42 | 0.897 |
| Orbitofrontal cortex (middle) (L) | 0.048 ± 0.062 | 0.029 ± 0.038 | 0.025 ± 0.042 | 1.02 | 0.841 |
| Orbitofrontal cortex (middle) (R) | 0.041 ± 0.033 | 0.039 ± 0.035 | 0.042 ± 0.063 | 0.01 | 0.992 |
| Inferior frontal gyrus (operculuar) (L) | 0.034 ± 0.044 | 0.026 ± 0.024 | 0.059 ± 0.061 | 2.21 | 0.784 |
| Inferior frontal gyrus (opercular) (R) | 0.031 ± 0.038 | 0.022 ± 0.027 | 0.037 ± 0.035 | 0.76 | 0.841 |
| Inferior frontal gyrus (triangular) (L) | 0.022 ± 0.026 | 0.026 ± 0.044 | 0.032 ± 0.027 | 0.35 | 0.914 |
| Inferior frontal gyrus (triangular) (R) | 0.050 ± 0.055 | 0.035 ± 0.041 | 0.017 ± 0.024 | 2.29 | 0.784 |
| Inferior frontal gyrus (orbitalis) (L) | 0.040 ± 0.049 | 0.031 ± 0.038 | 0.025 ± 0.036 | 0.52 | 0.859 |
| Inferior frontal gyrus (orbitalis) (R) | 0.031 ± 0.033 | 0.016 ± 0.014 | 0.017 ± 0.024 | 1.69 | 0.841 |
| Rolandic operculum (L) | 0.023 ± 0.031 | 0.030 ± 0.058 | 0.032 ± 0.050 | 0.14 | 0.941 |
| Rolandic operculum (R) | 0.014 ± 0.018 | 0.026 ± 0.034 | 0.026 ± 0.037 | 0.67 | 0.859 |
| Supplementary motor area (L) | 0.031 ± 0.053 | 0.030 ± 0.056 | 0.041 ± 0.043 | 0.22 | 0.931 |
| Supplementary motor area (R) | 0.047 ± 0.045 | 0.025 ± 0.027 | 0.047 ± 0.049 | 1.34 | 0.841 |
| Olfactory (L) | 0.045 ± 0.061 | 0.029 ± 0.029 | 0.080 ± 0.070 | 3.28 | 0.604 |
| Olfactory (R) | 0.063 ± 0.062 | 0.048 ± 0.055 | 0.023 ± 0.031 | 2.42 | 0.784 |
| Dorsomedial PFC (L) | 0.022 ± 0.030 | 0.052 ± 0.065 | 0.023 ± 0.041 | 1.94 | 0.841 |
| Dorsomedial PFC (R) | 0.033 ± 0.048 | 0.025 ± 0.031 | 0.056 ± 0.093 | 0.98 | 0.841 |
| Ventromedial PFC (L) | 0.016 ± 0.022 | 0.028 ± 0.051 | 0.029 ± 0.038 | 0.57 | 0.859 |
| Ventromedial PFC (R) | 0.054 ± 0.083 | 0.022 ± 0.032 | 0.041 ± 0.054 | 1.11 | 0.841 |
| Rectus gyrus (L) | 0.022 ± 0.029 | 0.039 ± 0.034 | 0.068 ± 0.069 | 3.63 | 0.604 |
| Rectus gyrus (R) | 0.007 ± 0.011 | 0.048 ± 0.036 | 0.031 ± 0.027 | 8.67 | 0.090 |
| Insula (L) | 0.015 ± 0.019 | 0.024 ± 0.034 | 0.027 ± 0.049 | 0.43 | 0.897 |
| Insula (R) | 0.009 ± 0.011 | 0.020 ± 0.018 | 0.016 ± 0.028 | 1.18 | 0.841 |
| Ventral ACC (L) | 0.013 ± 0.016 | 0.016 ± 0.015 | 0.030 ± 0.066 | 0.75 | 0.841 |
| Ventral ACC (R) | 0.031 ± 0.030 | 0.017 ± 0.028 | 0.026 ± 0.046 | 0.62 | 0.859 |
| Dorsal ACC (L) | 0.035 ± 0.053 | 0.063 ± 0.060 | 0.069 ± 0.084 | 1.09 | 0.841 |
| Dorsal ACC (R) | 0.051 ± 0.070 | 0.049 ± 0.057 | 0.045 ± 0.049 | 0.05 | 0.981 |
| Posterior cingulate cortex (L) | 0.035 ± 0.040 | 0.025 ± 0.040 | 0.049 ± 0.050 | 1.14 | 0.841 |
| Posterior cingulate cortex (R) | 0.027 ± 0.037 | 0.024 ± 0.025 | 0.031 ± 0.031 | 0.18 | 0.941 |
| Hippocampus (L) | 0.062 ± 0.090 | 0.022 ± 0.023 | 0.043 ± 0.045 | 1.71 | 0.841 |
| Hippocampus (R) | 0.045 ± 0.048 | 0.043 ± 0.042 | 0.030 ± 0.037 | 0.52 | 0.859 |
| Parahippocampal gyrus (L) | 0.031 ± 0.046 | 0.051 ± 0.069 | 0.025 ± 0.027 | 1.12 | 0.841 |
| Parahippocampal gyrus (R) | 0.036 ± 0.042 | 0.062 ± 0.064 | 0.041 ± 0.051 | 1.00 | 0.841 |
| Amygdala (L) | 0.031 ± 0.029 | 0.021 ± 0.053 | 0.022 ± 0.033 | 0.31 | 0.914 |
| Amygdala (R) | 0.048 ± 0.075 | 0.043 ± 0.056 | 0.019 ± 0.020 | 1.19 | 0.841 |
| Calcarine cortex (L) | 0.020 ± 0.051 | 0.021 ± 0.034 | 0.035 ± 0.044 | 0.57 | 0.859 |
| Calcarine cortex (R) | 0.045 ± 0.061 | 0.035 ± 0.039 | 0.052 ± 0.064 | 0.33 | 0.914 |
| Cuneus (L) | 0.031 ± 0.025 | 0.011 ± 0.015 | 0.025 ± 0.036 | 2.22 | 0.784 |
| Cuneus (R) | 0.035 ± 0.035 | 0.028 ± 0.038 | 0.038 ± 0.046 | 0.25 | 0.927 |
| Lingual gyrus (L) | 0.042 ± 0.073 | 0.039 ± 0.039 | 0.041 ± 0.047 | 0.01 | 0.992 |
| Lingual gyrus (R) | 0.036 ± 0.025 | 0.034 ± 0.045 | 0.043 ± 0.032 | 0.28 | 0.923 |
| Superior occipital gyrus (L) | 0.031 ± 0.039 | 0.033 ± 0.041 | 0.025 ± 0.031 | 0.22 | 0.931 |
| Superior occipital gyrus (R) | 0.022 ± 0.035 | 0.026 ± 0.036 | 0.021 ± 0.029 | 0.09 | 0.958 |
| Middle occipital gyrus (L) | 0.042 ± 0.052 | 0.046 ± 0.051 | 0.021 ± 0.035 | 1.24 | 0.841 |
| Middle occipital gyrus (R) | 0.041 ± 0.061 | 0.035 ± 0.053 | 0.025 ± 0.041 | 0.36 | 0.914 |
| Inferior occipital gyrus (L) | 0.027 ± 0.026 | 0.023 ± 0.023 | 0.040 ± 0.034 | 1.43 | 0.841 |
| Inferior occipital gyrus (R) | 0.076 ± 0.114 | 0.020 ± 0.021 | 0.047 ± 0.047 | 2.26 | 0.784 |
| Fusiform gyrus (L) | 0.028 ± 0.026 | 0.098 ± 0.091 | 0.034 ± 0.034 | 6.60 | 0.135 |
| Fusiform gyrus (R) | 0.043 ± 0.039 | 0.037 ± 0.039 | 0.034 ± 0.052 | 0.17 | 0.941 |
| Postcentral gyrus (L) | 0.020 ± 0.037 | 0.030 ± 0.028 | 0.021 ± 0.022 | 0.50 | 0.859 |
| Postcentral gyrus (R) | 0.048 ± 0.072 | 0.022 ± 0.026 | 0.027 ± 0.025 | 1.29 | 0.841 |
| Superior parietal lobule (L) | 0.028 ± 0.039 | 0.029 ± 0.031 | 0.042 ± 0.033 | 0.77 | 0.841 |
| Superior parietal lobule (R) | 0.042 ± 0.058 | 0.025 ± 0.032 | 0.044 ± 0.050 | 0.72 | 0.841 |
| Inferior parietal lobule (L) | 0.015 ± 0.016 | 0.012 ± 0.014 | 0.033 ± 0.047 | 2.29 | 0.784 |
| Inferior parietal lobule (R) | 0.017 ± 0.018 | 0.057 ± 0.046 | 0.064 ± 0.063 | 4.49 | 0.510 |
| Supramarginal gyrus (L) | 0.033 ± 0.052 | 0.057 ± 0.078 | 0.036 ± 0.038 | 0.73 | 0.841 |
| Supramarginal gyrus (R) | 0.017 ± 0.034 | 0.020 ± 0.030 | 0.025 ± 0.026 | 0.25 | 0.927 |
| Angular gyrus (L) | 0.020 ± 0.034 | 0.023 ± 0.022 | 0.041 ± 0.054 | 1.27 | 0.841 |
| Angular gyrus (R) | 0.020 ± 0.021 | 0.027 ± 0.030 | 0.046 ± 0.069 | 1.34 | 0.841 |
| Precuneus (L) | 0.018 ± 0.024 | 0.015 ± 0.021 | 0.019 ± 0.038 | 0.11 | 0.949 |
| Precuneus (R) | 0.027 ± 0.027 | 0.012 ± 0.020 | 0.031 ± 0.034 | 2.11 | 0.804 |
| Paracentral lobule (L) | 0.051 ± 0.049 | 0.037 ± 0.044 | 0.074 ± 0.096 | 1.15 | 0.841 |
| Paracentral lobule (R) | 0.019 ± 0.019 | 0.024 ± 0.022 | 0.048 ± 0.071 | 1.81 | 0.841 |
| Caudate (L) | 0.050 ± 0.047 | 0.017 ± 0.023 | 0.028 ± 0.041 | 2.79 | 0.784 |
| Caudate (R) | 0.089 ± 0.091 | 0.037 ± 0.043 | 0.038 ± 0.039 | 3.47 | 0.604 |
| Putamen (L) | 0.036 ± 0.047 | 0.039 ± 0.065 | 0.022 ± 0.026 | 0.52 | 0.859 |
| Putamen (R) | 0.017 ± 0.025 | 0.033 ± 0.058 | 0.042 ± 0.071 | 0.79 | 0.841 |
| Pallidum (L) | 0.044 ± 0.035 | 0.042 ± 0.066 | 0.038 ± 0.045 | 0.04 | 0.981 |
| Pallidum (R) | 0.019 ± 0.024 | 0.046 ± 0.053 | 0.029 ± 0.034 | 1.92 | 0.841 |
| Thalamus (L) | 0.061 ± 0.073 | 0.032 ± 0.037 | 0.033 ± 0.039 | 1.45 | 0.841 |
| Thalamus (R) | 0.049 ± 0.056 | 0.054 ± 0.097 | 0.030 ± 0.036 | 0.52 | 0.859 |
| Heschl's gyrus (L) | 0.033 ± 0.039 | 0.032 ± 0.048 | 0.026 ± 0.026 | 0.13 | 0.941 |
| Heschl's gyrus (R) | 0.056 ± 0.071 | 0.056 ± 0.068 | 0.029 ± 0.045 | 0.90 | 0.841 |
| Superior temporal gyrus (L) | 0.031 ± 0.041 | 0.031 ± 0.035 | 0.025 ± 0.043 | 0.14 | 0.941 |
| Superior temporal gyrus (R) | 0.025 ± 0.023 | 0.022 ± 0.037 | 0.059 ± 0.061 | 3.37 | 0.604 |
| Temporal pole (superior) (L) | 0.043 ± 0.078 | 0.027 ± 0.030 | 0.036 ± 0.056 | 0.30 | 0.914 |
| Temporal pole (superior) (R) | 0.026 ± 0.040 | 0.041 ± 0.063 | 0.021 ± 0.028 | 0.73 | 0.841 |
| Middle temporal gyrus (L) | 0.030 ± 0.035 | 0.024 ± 0.048 | 0.043 ± 0.042 | 0.80 | 0.841 |
| Middle temporal gyrus (R) | 0.048 ± 0.051 | 0.029 ± 0.035 | 0.049 ± 0.057 | 0.81 | 0.841 |
| Temporal pole (middle) (L) | 0.062 ± 0.060 | 0.040 ± 0.069 | 0.040 ± 0.044 | 0.72 | 0.841 |
| Temporal pole (middle) (R) | 0.049 ± 0.053 | 0.033 ± 0.040 | 0.046 ± 0.073 | 0.32 | 0.914 |
| Inferior temporal gyrus (L) | 0.052 ± 0.067 | 0.037 ± 0.034 | 0.042 ± 0.054 | 0.32 | 0.914 |
| Inferior temporal gyrus (R) | 0.044 ± 0.038 | 0.043 ± 0.047 | 0.027 ± 0.064 | 0.52 | 0.859 |

*^a^*Corrected *P* was obtained by Benjamini-Hochberg procedure to correct multiple comparisons.

Abbreviation: ACC, anterior cingulate cortex; ADHD, attention-deficit/hyperactivity disorder; L, left; mADHD, mild symptom ADHD; PFC, prefrontal cortex; R, right; sADHD, severe symptom ADHD; SD, standard deviation; TDC, typically developing controls.
