# Supplementary material for: Novel subgroups of attention-deficit/hyperactivity disorder identified by topological data analysis and their functional network modular organizations
Source: PLoS One. 2017 Aug 22;12(8):e0182603. doi: 10.1371/journal.pone.0182603 (PMC5567504; doi:10.1371/journal.pone.0182603)
Supplement: S4 Table — (DOCX) [file pone.0182603.s006.docx]

**S4 Table**. Mean values of PageRank for each mADHD and sADHD subgroup and its statistical comparison using analysis of variance

| Anatomical Region | TDC | mADHD | sADHD | Analysis of Variance | |
| --- | --- | --- | --- | --- | --- |
|  | Mean ± SD | Mean ± SD | Mean ± SD | *F*_2,42_ | Corrected *P^a^* |
| Precentral gyrus (L) | 0.0100 ± 0.0014 | 0.0091 ± 0.0021 | 0.0093 ± 0.0017 | 1.29 | 0.644 |
| Precentral gyrus (R) | 0.0109 ± 0.0016 | 0.0105 ± 0.0014 | 0.0115 ± 0.0020 | 1.48 | 0.617 |
| Superior frontal gyrus (L) | 0.0111 ± 0.0023 | 0.0101 ± 0.0010 | 0.0099 ± 0.0016 | 2.19 | 0.524 |
| Superior frontal gyrus (R) | 0.0092 ± 0.0016 | 0.0095 ± 0.0016 | 0.0099 ± 0.0016 | 0.74 | 0.755 |
| Orbitofrontal cortex (superior) (L) | 0.0127 ± 0.0020 | 0.0111 ± 0.0016 | 0.0119 ± 0.0019 | 2.81 | 0.422 |
| Orbitofrontal cortex (superior) (R) | 0.0109 ± 0.0018 | 0.0106 ± 0.0018 | 0.0117 ± 0.0019 | 1.60 | 0.617 |
| Dorsolateral PFC (L) | 0.0097 ± 0.0017 | 0.0094 ± 0.0017 | 0.0102 ± 0.0013 | 1.03 | 0.707 |
| Dorsolateral PFC (R) | 0.0095 ± 0.0012 | 0.0093 ± 0.0012 | 0.0102 ± 0.0015 | 2.21 | 0.524 |
| Orbitofrontal cortex (middle) (L) | 0.0105 ± 0.0021 | 0.0104 ± 0.0015 | 0.0116 ± 0.0016 | 2.11 | 0.524 |
| Orbitofrontal cortex (middle) (R) | 0.0108 ± 0.0017 | 0.0104 ± 0.0014 | 0.0117 ± 0.0020 | 2.28 | 0.524 |
| Inferior frontal gyrus (operculuar) (L) | 0.0106 ± 0.0011 | 0.0116 ± 0.0023 | 0.0114 ± 0.0013 | 1.51 | 0.617 |
| Inferior frontal gyrus (opercular) (R) | 0.0116 ± 0.0015 | 0.0118 ± 0.0018 | 0.0115 ± 0.0013 | 0.08 | 0.932 |
| Inferior frontal gyrus (triangular) (L) | 0.0109 ± 0.0014 | 0.0108 ± 0.0017 | 0.0113 ± 0.0013 | 0.42 | 0.809 |
| Inferior frontal gyrus (triangular) (R) | 0.0110 ± 0.0013 | 0.0111 ± 0.0017 | 0.0118 ± 0.0015 | 1.56 | 0.617 |
| Inferior frontal gyrus (orbitalis) (L) | 0.0120 ± 0.0022 | 0.0115 ± 0.0020 | 0.0127 ± 0.0012 | 1.55 | 0.617 |
| Inferior frontal gyrus (orbitalis) (R) | 0.0129 ± 0.0018 | 0.0127 ± 0.0016 | 0.0137 ± 0.0017 | 1.41 | 0.638 |
| Rolandic operculum (L) | 0.0128 ± 0.0014 | 0.0137 ± 0.0011 | 0.0128 ± 0.0018 | 1.72 | 0.617 |
| Rolandic operculum (R) | 0.0128 ± 0.0013 | 0.0134 ± 0.0012 | 0.0129 ± 0.0016 | 0.68 | 0.755 |
| Supplementary motor area (L) | 0.0107 ± 0.0017 | 0.0106 ± 0.0014 | 0.0104 ± 0.0012 | 0.19 | 0.895 |
| Supplementary motor area (R) | 0.0097 ± 0.0019 | 0.0096 ± 0.0016 | 0.0104 ± 0.0013 | 1.14 | 0.675 |
| Olfactory (L) | 0.0111 ± 0.0021 | 0.0104 ± 0.0019 | 0.0104 ± 0.0020 | 0.69 | 0.755 |
| Olfactory (R) | 0.0094 ± 0.0021 | 0.0094 ± 0.0014 | 0.0102 ± 0.0019 | 0.90 | 0.754 |
| Dorsomedial PFC (L) | 0.0130 ± 0.0016 | 0.0115 ± 0.0015 | 0.0118 ± 0.0020 | 3.18 | 0.422 |
| Dorsomedial PFC (R) | 0.0118 ± 0.0017 | 0.0109 ± 0.0014 | 0.0116 ± 0.0017 | 1.49 | 0.617 |
| Ventromedial PFC (L) | 0.0139 ± 0.0018 | 0.0126 ± 0.0010 | 0.0129 ± 0.0017 | 3.04 | 0.422 |
| Ventromedial PFC (R) | 0.0135 ± 0.0014 | 0.0122 ± 0.0016 | 0.0130 ± 0.0015 | 2.67 | 0.429 |
| Rectus gyrus (L) | 0.0136 ± 0.0018 | 0.0122 ± 0.0016 | 0.0127 ± 0.0017 | 2.76 | 0.422 |
| Rectus gyrus (R) | 0.0130 ± 0.0015 | 0.0119 ± 0.0020 | 0.0125 ± 0.0020 | 1.35 | 0.644 |
| Insula (L) | 0.0131 ± 0.0017 | 0.0131 ± 0.0010 | 0.0128 ± 0.0017 | 0.19 | 0.895 |
| Insula (R) | 0.0144 ± 0.0009 | 0.0144 ± 0.0012 | 0.0141 ± 0.0012 | 0.62 | 0.760 |
| Ventral ACC (L) | 0.0122 ± 0.0015 | 0.0118 ± 0.0013 | 0.0124 ± 0.0016 | 0.80 | 0.754 |
| Ventral ACC (R) | 0.0113 ± 0.0020 | 0.0120 ± 0.0011 | 0.0129 ± 0.0016 | 3.75 | 0.288 |
| Dorsal ACC (L) | 0.0099 ± 0.0018 | 0.0094 ± 0.0016 | 0.0100 ± 0.0015 | 0.51 | 0.771 |
| Dorsal ACC (R) | 0.0105 ± 0.0015 | 0.0097 ± 0.0016 | 0.0103 ± 0.0011 | 1.14 | 0.675 |
| Posterior cingulate cortex (L) | 0.0123 ± 0.0018 | 0.0111 ± 0.0012 | 0.0096 ± 0.0012 | 13.67 | <0.001 |
| Posterior cingulate cortex (R) | 0.0105 ± 0.0017 | 0.0096 ± 0.0012 | 0.0082 ± 0.0011 | 10.95 | <0.001 |
| Hippocampus (L) | 0.0097 ± 0.0017 | 0.0101 ± 0.0017 | 0.0094 ± 0.0019 | 0.68 | 0.755 |
| Hippocampus (R) | 0.0088 ± 0.0021 | 0.0094 ± 0.0015 | 0.0087 ± 0.0015 | 0.61 | 0.760 |
| Parahippocampal gyrus (L) | 0.0105 ± 0.0016 | 0.0096 ± 0.0018 | 0.0102 ± 0.0018 | 1.15 | 0.675 |
| Parahippocampal gyrus (R) | 0.0107 ± 0.0016 | 0.0110 ± 0.0020 | 0.0107 ± 0.0013 | 0.15 | 0.895 |
| Amygdala (L) | 0.0119 ± 0.0017 | 0.0129 ± 0.0018 | 0.0121 ± 0.0018 | 1.32 | 0.644 |
| Amygdala (R) | 0.0129 ± 0.0016 | 0.0130 ± 0.0017 | 0.0124 ± 0.0017 | 0.64 | 0.760 |
| Calcarine cortex (L) | 0.0104 ± 0.0012 | 0.0103 ± 0.0011 | 0.0101 ± 0.0011 | 0.20 | 0.895 |
| Calcarine cortex (R) | 0.0101 ± 0.0014 | 0.0107 ± 0.0011 | 0.0110 ± 0.0012 | 1.94 | 0.585 |
| Cuneus (L) | 0.0107 ± 0.0014 | 0.0103 ± 0.0012 | 0.0107 ± 0.0011 | 0.56 | 0.768 |
| Cuneus (R) | 0.0104 ± 0.0011 | 0.0109 ± 0.0013 | 0.0110 ± 0.0015 | 1.04 | 0.707 |
| Lingual gyrus (L) | 0.0101 ± 0.0015 | 0.0115 ± 0.0018 | 0.0114 ± 0.0014 | 3.76 | 0.288 |
| Lingual gyrus (R) | 0.0107 ± 0.0015 | 0.0116 ± 0.0015 | 0.0113 ± 0.0015 | 1.49 | 0.617 |
| Superior occipital gyrus (L) | 0.0102 ± 0.0015 | 0.0112 ± 0.0013 | 0.0111 ± 0.0011 | 2.81 | 0.422 |
| Superior occipital gyrus (R) | 0.0097 ± 0.0011 | 0.0103 ± 0.0013 | 0.0098 ± 0.0008 | 1.02 | 0.707 |
| Middle occipital gyrus (L) | 0.0107 ± 0.0014 | 0.0112 ± 0.0008 | 0.0110 ± 0.0010 | 0.93 | 0.754 |
| Middle occipital gyrus (R) | 0.0104 ± 0.0017 | 0.0111 ± 0.0011 | 0.0105 ± 0.0009 | 1.26 | 0.648 |
| Inferior occipital gyrus (L) | 0.0104 ± 0.0012 | 0.0108 ± 0.0015 | 0.0103 ± 0.0011 | 0.53 | 0.771 |
| Inferior occipital gyrus (R) | 0.0096 ± 0.0016 | 0.0102 ± 0.0012 | 0.0098 ± 0.0011 | 0.76 | 0.755 |
| Fusiform gyrus (L) | 0.0112 ± 0.0012 | 0.0114 ± 0.0019 | 0.0109 ± 0.0020 | 0.36 | 0.839 |
| Fusiform gyrus (R) | 0.0102 ± 0.0017 | 0.0113 ± 0.0016 | 0.0106 ± 0.0017 | 1.75 | 0.617 |
| Postcentral gyrus (L) | 0.0113 ± 0.0014 | 0.0112 ± 0.0013 | 0.0109 ± 0.0017 | 0.34 | 0.845 |
| Postcentral gyrus (R) | 0.0116 ± 0.0018 | 0.0113 ± 0.0015 | 0.0119 ± 0.0022 | 0.41 | 0.809 |
| Superior parietal lobule (L) | 0.0092 ± 0.0012 | 0.0095 ± 0.0010 | 0.0089 ± 0.0016 | 0.79 | 0.754 |
| Superior parietal lobule (R) | 0.0100 ± 0.0010 | 0.0095 ± 0.0010 | 0.0095 ± 0.0017 | 0.81 | 0.754 |
| Inferior parietal lobule (L) | 0.0098 ± 0.0016 | 0.0099 ± 0.0012 | 0.0096 ± 0.0010 | 0.16 | 0.895 |
| Inferior parietal lobule (R) | 0.0102 ± 0.0017 | 0.0102 ± 0.0012 | 0.0098 ± 0.0016 | 0.33 | 0.845 |
| Supramarginal gyrus (L) | 0.0116 ± 0.0019 | 0.0114 ± 0.0018 | 0.0110 ± 0.0014 | 0.59 | 0.760 |
| Supramarginal gyrus (R) | 0.0114 ± 0.0020 | 0.0109 ± 0.0014 | 0.0109 ± 0.0009 | 0.42 | 0.809 |
| Angular gyrus (L) | 0.0120 ± 0.0021 | 0.0105 ± 0.0013 | 0.0101 ± 0.0015 | 5.72 | 0.108 |
| Angular gyrus (R) | 0.0109 ± 0.0017 | 0.0105 ± 0.0015 | 0.0102 ± 0.0019 | 0.70 | 0.755 |
| Precuneus (L) | 0.0102 ± 0.0012 | 0.0096 ± 0.0015 | 0.0090 ± 0.0007 | 4.23 | 0.236 |
| Precuneus (R) | 0.0097 ± 0.0011 | 0.0093 ± 0.0016 | 0.0091 ± 0.0010 | 0.86 | 0.754 |
| Paracentral lobule (L) | 0.0084 ± 0.0016 | 0.0086 ± 0.0014 | 0.0094 ± 0.0018 | 1.73 | 0.617 |
| Paracentral lobule (R) | 0.0084 ± 0.0014 | 0.0088 ± 0.0014 | 0.0095 ± 0.0013 | 2.51 | 0.465 |
| Caudate (L) | 0.0082 ± 0.0014 | 0.0097 ± 0.0013 | 0.0099 ± 0.0014 | 7.39 | 0.045 |
| Caudate (R) | 0.0079 ± 0.0016 | 0.0098 ± 0.0014 | 0.0100 ± 0.0017 | 8.65 | 0.030 |
| Putamen (L) | 0.0132 ± 0.0012 | 0.0134 ± 0.0017 | 0.0131 ± 0.0014 | 0.17 | 0.895 |
| Putamen (R) | 0.0139 ± 0.0008 | 0.0137 ± 0.0016 | 0.0134 ± 0.0016 | 0.55 | 0.768 |
| Pallidum (L) | 0.0116 ± 0.0012 | 0.0122 ± 0.0014 | 0.0121 ± 0.0016 | 0.79 | 0.754 |
| Pallidum (R) | 0.0130 ± 0.0008 | 0.0130 ± 0.0019 | 0.0127 ± 0.0011 | 0.23 | 0.895 |
| Thalamus (L) | 0.0083 ± 0.0015 | 0.0102 ± 0.0018 | 0.0100 ± 0.0019 | 5.11 | 0.150 |
| Thalamus (R) | 0.0093 ± 0.0017 | 0.0099 ± 0.0015 | 0.0105 ± 0.0016 | 2.15 | 0.524 |
| Heschl's gyrus (L) | 0.0120 ± 0.0009 | 0.0125 ± 0.0021 | 0.0127 ± 0.0016 | 0.61 | 0.760 |
| Heschl's gyrus (R) | 0.0122 ± 0.0009 | 0.0124 ± 0.0023 | 0.0125 ± 0.0013 | 0.13 | 0.895 |
| Superior temporal gyrus (L) | 0.0132 ± 0.0014 | 0.0139 ± 0.0017 | 0.0133 ± 0.0016 | 0.82 | 0.754 |
| Superior temporal gyrus (R) | 0.0131 ± 0.0012 | 0.0133 ± 0.0020 | 0.0129 ± 0.0019 | 0.21 | 0.895 |
| Temporal pole (superior) (L) | 0.0139 ± 0.0017 | 0.0138 ± 0.0022 | 0.0137 ± 0.0014 | 0.02 | 0.985 |
| Temporal pole (superior) (R) | 0.0141 ± 0.0012 | 0.0144 ± 0.0020 | 0.0141 ± 0.0017 | 0.14 | 0.895 |
| Middle temporal gyrus (L) | 0.0117 ± 0.0013 | 0.0121 ± 0.0017 | 0.0110 ± 0.0019 | 1.77 | 0.617 |
| Middle temporal gyrus (R) | 0.0114 ± 0.0016 | 0.0113 ± 0.0014 | 0.0101 ± 0.0020 | 2.75 | 0.422 |
| Temporal pole (middle) (L) | 0.0122 ± 0.0023 | 0.0122 ± 0.0022 | 0.0119 ± 0.0017 | 0.14 | 0.895 |
| Temporal pole (middle) (R) | 0.0120 ± 0.0015 | 0.0125 ± 0.0016 | 0.0109 ± 0.0014 | 4.78 | 0.167 |
| Inferior temporal gyrus (L) | 0.0109 ± 0.0016 | 0.0105 ± 0.0014 | 0.0100 ± 0.0016 | 1.31 | 0.644 |
| Inferior temporal gyrus (R) | 0.0099 ± 0.0017 | 0.0102 ± 0.0015 | 0.0104 ± 0.0016 | 0.50 | 0.771 |

*^a^*Corrected *P* was obtained by Benjamini-Hochberg procedure to correct multiple comparisons.

Abbreviation: ACC, anterior cingulate cortex; ADHD, attention-deficit/hyperactivity disorder; L, left; mADHD, mild symptom ADHD; PFC, prefrontal cortex; R, right; sADHD, severe symptom ADHD; SD, standard deviation; TDC, typically developing controls.
