# Supplementary material for: Novel subgroups of attention-deficit/hyperactivity disorder identified by topological data analysis and their functional network modular organizations
Source: PLoS One. 2017 Aug 22;12(8):e0182603. doi: 10.1371/journal.pone.0182603 (PMC5567504; doi:10.1371/journal.pone.0182603)
Supplement: S5 Table — Modularity optimization analyses were conducted for the principal data set. (DOCX) [file pone.0182603.s007.docx]

S5 Table. Functional modular organization for each subgroup. Modularity optimization analyses were conducted for the principal data set

|  | Typically Developing Controls | Mild ADHD | Severe ADHD |
| --- | --- | --- | --- |
| **DMN,** | Superior frontal gyrus (L) | Superior frontal gyrus (L) | Superior frontal gyrus (L) |
| Default | Orbitofrontal cortex (superior) (L) | Orbitofrontal cortex (superior) (L) | Orbitofrontal cortex (superior) (L/R) |
| Mode | Olfactory (L/R) | Olfactory (L/R) | Orbitofrontal cortex (middle) (L/R) |
| Network |  |  | Inferior frontal gyrus (orbitalis) (L/R) |
|  |  |  | Olfactory (L/R) |
|  | Dorsomedial PFC (L/R) | Dorsomedial PFC (L/R) | Dorsomedial PFC (L/R) |
|  | Ventromedial PFC (L/R) | Ventromedial PFC (L/R) | Ventromedial PFC (L/R) |
|  | Rectus gyrus (L/R) | Rectus gyrus (L/R) | Rectus gyrus (L/R) |
|  | Ventral ACC (L/R) | Ventral ACC (L/R) | Ventral ACC (L/R) |
|  | Posterior cingulate cortex (L/R) | Posterior cingulate cortex (L/R) | Posterior cingulate cortex (L/R) |
|  | Hippocampus (L/R) |  |  |
|  | Parahippocampal gyrus (L/R) |  |  |
|  | Angular gyrus (L) | Angular gyrus (L/R) | Angular gyrus (L) |
|  | Middle temporal gyrus (L/R) | Middle temporal gyrus (L/R) | Temporal pole (superior) (L) |
|  | Temporal pole (middle) (L/R) | Temporal pole (middle) (L/R) | Middle temporal gyrus (L/R) |
|  |  | Superior frontal gyrus (R) | Temporal pole (middle) (L/R) |
|  |  |  | Inferior temporal gyrus (L/R) |
| **VN,** | Calcarine cortex (L/R) | Calcarine cortex (L/R) | Calcarine cortex (L/R) |
| Visual | Cuneus (L/R) | Cuneus (L/R) | Cuneus (L/R) |
| Network | Lingual gyrus (L/R) | Lingual gyrus (L/R) | Lingual gyrus (L/R) |
|  | Superior occipital gyrus (L/R) | Superior occipital gyrus (L/R) | Superior occipital gyrus (L/R) |
|  | Middle occipital gyrus (L/R) | Middle occipital gyrus (L/R) | Middle occipital gyrus (L/R) |
|  | Inferior occipital gyrus (L/R) | Inferior occipital gyrus (L/R) | Inferior occipital gyrus (L/R) |
|  | Fusiform gyrus (L/R) | Fusiform gyrus (L/R) | Fusiform gyrus (L/R) |
|  | Superior parietal lobule (L/R) | Superior parietal lobule (L/R) | Superior parietal lobule (L/R) |
|  | Precuneus (L/R) | Precuneus (L/R) | Precuneus (L/R) |
| **SN,** | Precentral gyrus (L/R) | Precentral gyrus (L/R) | Precentral gyrus (L/R) |
| Salience | Rolandic operculum (L/R) | Rolandic operculum (L/R) | Rolandic operculum (L/R) |
| Network | Supplementary motor area (L/R) | Supplementary motor area (L/R) | Supplementary motor area (L/R) |
|  | Insula (L/R) | Insula (L/R) | Insula (L/R) |
|  | Dorsal ACC (L/R) | Dorsal ACC (L/R) | Dorsal ACC (L/R) |
|  | Amygdala (L/R) |  |  |
|  | Postcentral gyrus (L/R) | Postcentral gyrus (L/R) | Postcentral gyrus (L/R) |
|  | Supramarginal gyrus (L/R) | Supramarginal gyrus (L/R) | Supramarginal gyrus (L/R) |
|  | Paracentral lobule (L/R) | Paracentral lobule (L/R) | Paracentral lobule (L/R) |
|  | Putamen (L/R) | Putamen (R) | Putamen (R) |
|  | Pallidum (L/R) |  |  |
|  | Heschl's gyrus (L/R) | Heschl's gyrus (L/R) | Heschl's gyrus (L/R) |
|  | Superior temporal gyrus (L/R) | Superior temporal gyrus (L/R) | Superior temporal gyrus (L/R) |
|  | Temporal pole (superior) (L/R) | Temporal pole (superior) (L/R) | Temporal pole (superior) (R) |
| **ECN,** | Superior frontal gyrus (R) |  | Superior frontal gyrus (R) |
| Executive | Orbitofrontal cortex (superior) (R) | Orbitofrontal cortex (superior) (R) | |
| Control | Dorsolateral PFC (L/R) | Dorsolateral PFC (L/R) | Dorsolateral PFC (L/R) |
| Network | Orbitofrontal cortex (middle) (L/R) | Orbitofrontal cortex (middle) (L/R) | |
|  | Inferior frontal gyrus (opercular) (L/R) | Inferior frontal gyrus (opercular) (L/R) | Inferior frontal gyrus (opercular) (L/R) |
|  | Inferior frontal gyrus (triangular) (L/R) | Inferior frontal gyrus (triangular) (L/R) | Inferior frontal gyrus (triangular) (L/R) |
|  | Inferior frontal gyrus (orbitalis) (L/R) | Inferior frontal gyrus (orbitalis) (L/R) | |
|  | Inferior parietal lobule (L/R) | Inferior parietal lobule (L/R) | Inferior parietal lobule (L/R) |
|  | Angular gyrus (R) |  | Angular gyrus (R) |
|  | Inferior temporal gyrus (L/R) | Inferior temporal gyrus (L/R) |  |
| **BGN,** | Caudate (L/R) | Caudate (L/R) | Caudate (L/R) |
| Basal | Thalamus (L/R) | Thalamus (L/R) | Thalamus (L/R) |
| Ganglia |  | Hippocampus (L/R) | Hippocampus (L/R) |
| Network |  | Parahippocampal gyrus (L/R) | Parahippocampal gyrus (L/R) |
|  |  | Amygdala (L/R) | Amygdala (L/R) |
|  |  | Putamen (L) | Putamen (L) |
|  |  | Pallidum (L/R) | Pallidum (L/R) |

Abbreviation: ACC, anterior cingulate cortex; ADHD, attention-deficit/hyperactivity disorder; L, left; PFC, prefrontal cortex; R, right.
