# Supplementary material for: Novel subgroups of attention-deficit/hyperactivity disorder identified by topological data analysis and their functional network modular organizations
Source: PLoS One. 2017 Aug 22;12(8):e0182603. doi: 10.1371/journal.pone.0182603 (PMC5567504; doi:10.1371/journal.pone.0182603)
Supplement: S6 Table — (DOCX) [file pone.0182603.s008.docx]

**S6 Table**. Demographic and clinical characteristics of the inattentive and combined subtypes

| Variable | Inattentive type | Combined type | *T* | *P*-value |
| --- | --- | --- | --- | --- |
|  | Mean ± SD | Mean ± SD |  |  |
| Age | 12.82 ± 1.95 | 11.80 ± 1.76 | 2.18 | 0.033 |
| Intelligence quotient (IQ) | |  |  |  |
| Full-scale IQ | 102.53 ± 11.55 | 109.44 ± 12.28 | -2.34 | 0.022 |
| Verbal IQ | 107.75 ± 14.10 | 115.07 ± 17.77 | -1.88 | 0.065 |
| Performance IQ | 95.73 ± 12.96 | 101.00 ± 13.07 | -1.63 | 0.108 |
| Symptom severity |  |  |  |  |
| ADHD index | 47.05 ± 7.65 | 56.70 ± 7.36 | -5.15 | <0.001 |
| Inattentive | 27.45 ± 3.44 | 29.30 ± 3.80 | -2.07 | 0.043 |
| Hyper/impulsivity | 19.60 ± 5.43 | 27.41 ± 4.98 | -5.96 | <0.001 |

Mean and SD were acquired from the principal dataset.

Abbreviation: ADHD, attention deficit hyperactivity disorder; TDC, typically developing control; SD, standard deviation.
