# Supplementary material for: Novel subgroups of attention-deficit/hyperactivity disorder identified by topological data analysis and their functional network modular organizations
Source: PLoS One. 2017 Aug 22;12(8):e0182603. doi: 10.1371/journal.pone.0182603 (PMC5567504; doi:10.1371/journal.pone.0182603)
Supplement: S7 Table — The statistical significance of the intra-module and inter-module FCD were summarized. (DOCX) [file pone.0182603.s009.docx]

**S7 Table**. Two-sample t-test of the functional connectivity density (FCD) between the inattentive and combined subtypes. The statistical significance of the intra-module and inter-module FCD were summarized

| Functional module | Inattentive type | Combined type | *T* | Corrected *P^a^* |
| --- | --- | --- | --- | --- |
|  | Mean ± SD | Mean ± SD |  |  |
| intra-module FCD |  |  |  |  |
| Default Mode Network (DMN) | 0.29 ± 0.09 | 0.26 ± 0.05 | 1.19 | 0.805 |
| Executive Control Network (ECN) | 0.27 ± 0.07 | 0.27 ± 0.08 | -0.26 | 0.810 |
| Salience Network (SN) | 0.34 ± 0.08 | 0.35 ± 0.09 | -0.46 | 0.805 |
| Visual Network (VN) | 0.39 ± 0.13 | 0.42 ± 0.10 | -0.90 | 0.805 |
| Basal Ganglia Network (BGN) | 0.34 ± 0.09 | 0.35 ± 0.10 | -0.43 | 0.805 |
| Inter-module FCD |  |  |  |  |
| DMN – ECN | 0.03 ± 0.07 | 0.05 ± 0.07 | -1.22 | 0.805 |
| DMN – SN | -0.09 ± 0.08 | -0.08 ± 0.06 | -0.24 | 0.810 |
| DMN – VN | -0.07 ± 0.07 | -0.06 ± 0.07 | -0.39 | 0.805 |
| DMN – BGN | 0.06 ± 0.06 | 0.04 ± 0.07 | 0.90 | 0.805 |
| ECN – SN | 0.00 ± 0.06 | -0.01 ± 0.08 | 0.70 | 0.805 |
| ECN – VN | -0.10 ± 0.07 | -0.07 ± 0.09 | -1.64 | 0.795 |
| ECN – BGN | 0.00 ± 0.06 | -0.01 ± 0.08 | 0.54 | 0.805 |
| SN – VN | -0.08 ± 0.08 | -0.12 ± 0.08 | 2.00 | 0.735 |
| SN – BGN | 0.03 ± 0.07 | 0.04 ± 0.11 | -0.45 | 0.805 |
| VN – BGN | -0.20 ± 0.09 | -0.19 ± 0.06 | -0.53 | 0.805 |

*^a^*Corrected *P* was obtained by Benjamini-Hochberg procedure to correct multiple comparisons.

Mean and SD were acquired from the principal dataset.

Abbreviation: ADHD, attention deficit hyperactivity disorder; mADHD, children with mild symptom ADHD; sADHD, children with severe symptom ADHD; SD, standard deviation.
