# Supplementary material for: Novel subgroups of attention-deficit/hyperactivity disorder identified by topological data analysis and their functional network modular organizations
Source: PLoS One. 2017 Aug 22;12(8):e0182603. doi: 10.1371/journal.pone.0182603 (PMC5567504; doi:10.1371/journal.pone.0182603)
Supplement: S8 Table — (DOCX) [file pone.0182603.s010.docx]

**S8 Table**. Mean values of degree centrality for each inattentive and combined subtype and its statistical comparison using two-sample t-test

| Anatomical Region | Inattentive type | Combined type | *T* | Corrected *P* |
| --- | --- | --- | --- | --- |
|  | Mean ± SD | Mean ± SD |  |  |
| Precentral gyrus (L) | 10.31 ± 2.92 | 9.61 ± 2.22 | 1.05 | 0.979 |
| Precentral gyrus (R) | 11.98 ± 2.40 | 12.07 ± 2.90 | -0.14 | 0.979 |
| Superior frontal gyrus (L) | 10.98 ± 2.60 | 10.56 ± 2.66 | 0.65 | 0.979 |
| Superior frontal gyrus (R) | 9.67 ± 2.56 | 10.17 ± 2.72 | -0.76 | 0.979 |
| Orbitofrontal cortex (superior) (L) | 13.06 ± 2.52 | 12.88 ± 2.84 | 0.28 | 0.979 |
| Orbitofrontal cortex (superior) (R) | 11.56 ± 2.61 | 12.04 ± 2.79 | -0.72 | 0.979 |
| Dorsolateral PFC (L) | 9.72 ± 2.66 | 10.19 ± 2.38 | -0.74 | 0.979 |
| Dorsolateral PFC (R) | 9.75 ± 2.32 | 10.16 ± 2.47 | -0.69 | 0.979 |
| Orbitofrontal cortex (middle) (L) | 10.91 ± 2.74 | 12.29 ± 2.81 | -2.01 | 0.750 |
| Orbitofrontal cortex (middle) (R) | 11.15 ± 2.34 | 11.72 ± 2.82 | -0.90 | 0.979 |
| Inferior frontal gyrus (operculuar) (L) | 13.00 ± 3.02 | 12.59 ± 2.49 | 0.57 | 0.979 |
| Inferior frontal gyrus (opercular) (R) | 12.93 ± 2.77 | 12.77 ± 2.36 | 0.24 | 0.979 |
| Inferior frontal gyrus (triangular) (L) | 12.25 ± 3.08 | 12.21 ± 3.03 | 0.06 | 0.979 |
| Inferior frontal gyrus (triangular) (R) | 12.55 ± 2.64 | 13.26 ± 2.93 | -1.02 | 0.979 |
| Inferior frontal gyrus (orbitalis) (L) | 14.25 ± 3.26 | 14.69 ± 3.48 | -0.53 | 0.979 |
| Inferior frontal gyrus (orbitalis) (R) | 15.14 ± 2.93 | 15.64 ± 3.11 | -0.66 | 0.979 |
| Rolandic operculum (L) | 15.45 ± 2.49 | 15.23 ± 2.85 | 0.34 | 0.979 |
| Rolandic operculum (R) | 15.08 ± 2.71 | 14.60 ± 2.88 | 0.69 | 0.979 |
| Supplementary motor area (L) | 10.91 ± 2.88 | 11.92 ± 2.66 | -1.44 | 0.979 |
| Supplementary motor area (R) | 11.07 ± 2.99 | 11.34 ± 2.26 | -0.39 | 0.979 |
| Olfactory (L) | 11.86 ± 2.62 | 12.10 ± 3.05 | -0.34 | 0.979 |
| Olfactory (R) | 10.77 ± 2.70 | 11.02 ± 3.15 | -0.36 | 0.979 |
| Dorsomedial PFC (L) | 12.41 ± 2.76 | 12.25 ± 2.44 | 0.24 | 0.979 |
| Dorsomedial PFC (R) | 12.29 ± 2.26 | 12.13 ± 2.48 | 0.27 | 0.979 |
| Ventromedial PFC (L) | 14.49 ± 2.27 | 14.20 ± 2.42 | 0.50 | 0.979 |
| Ventromedial PFC (R) | 14.00 ± 2.26 | 13.86 ± 2.71 | 0.23 | 0.979 |
| Rectus gyrus (L) | 14.78 ± 2.73 | 14.49 ± 2.48 | 0.45 | 0.979 |
| Rectus gyrus (R) | 14.22 ± 2.68 | 13.83 ± 2.69 | 0.59 | 0.979 |
| Insula (L) | 15.26 ± 2.20 | 15.71 ± 2.68 | -0.76 | 0.979 |
| Insula (R) | 17.06 ± 2.33 | 17.19 ± 2.49 | -0.22 | 0.979 |
| Ventral ACC (L) | 13.73 ± 2.84 | 14.77 ± 2.39 | -1.56 | 0.979 |
| Ventral ACC (R) | 13.48 ± 2.71 | 14.44 ± 2.59 | -1.45 | 0.979 |
| Dorsal ACC (L) | 10.55 ± 2.76 | 10.53 ± 2.85 | 0.03 | 0.979 |
| Dorsal ACC (R) | 10.87 ± 2.79 | 10.78 ± 1.92 | 0.15 | 0.979 |
| Posterior cingulate cortex (L) | 11.39 ± 2.08 | 10.03 ± 1.69 | 2.82 | 0.450 |
| Posterior cingulate cortex (R) | 9.46 ± 1.94 | 8.37 ± 1.79 | 2.32 | 0.720 |
| Hippocampus (L) | 9.98 ± 3.16 | 9.24 ± 2.20 | 1.06 | 0.979 |
| Hippocampus (R) | 8.87 ± 2.97 | 9.16 ± 2.38 | -0.42 | 0.979 |
| Parahippocampal gyrus (L) | 10.68 ± 3.18 | 10.66 ± 2.22 | 0.03 | 0.979 |
| Parahippocampal gyrus (R) | 11.76 ± 2.80 | 11.94 ± 2.63 | -0.26 | 0.979 |
| Amygdala (L) | 14.54 ± 3.57 | 14.35 ± 2.73 | 0.23 | 0.979 |
| Amygdala (R) | 14.58 ± 3.51 | 15.16 ± 3.06 | -0.70 | 0.979 |
| Calcarine cortex (L) | 10.08 ± 2.59 | 10.77 ± 2.36 | -1.11 | 0.979 |
| Calcarine cortex (R) | 10.74 ± 2.73 | 11.26 ± 2.35 | -0.80 | 0.979 |
| Cuneus (L) | 10.70 ± 2.01 | 11.01 ± 1.88 | -0.62 | 0.979 |
| Cuneus (R) | 11.10 ± 2.33 | 11.22 ± 2.55 | -0.20 | 0.979 |
| Lingual gyrus (L) | 11.99 ± 3.22 | 11.96 ± 2.82 | 0.03 | 0.979 |
| Lingual gyrus (R) | 12.12 ± 3.34 | 11.69 ± 2.53 | 0.57 | 0.979 |
| Superior occipital gyrus (L) | 11.68 ± 2.19 | 11.43 ± 2.08 | 0.47 | 0.979 |
| Superior occipital gyrus (R) | 10.47 ± 2.36 | 10.40 ± 1.57 | 0.15 | 0.979 |
| Middle occipital gyrus (L) | 11.46 ± 2.10 | 11.68 ± 1.75 | -0.45 | 0.979 |
| Middle occipital gyrus (R) | 10.91 ± 2.24 | 11.61 ± 1.84 | -1.35 | 0.979 |
| Inferior occipital gyrus (L) | 10.97 ± 2.26 | 10.89 ± 2.07 | 0.15 | 0.979 |
| Inferior occipital gyrus (R) | 10.99 ± 2.58 | 10.20 ± 2.29 | 1.30 | 0.979 |
| Fusiform gyrus (L) | 12.60 ± 3.21 | 11.23 ± 2.74 | 1.81 | 0.750 |
| Fusiform gyrus (R) | 12.11 ± 2.55 | 10.86 ± 2.58 | 1.95 | 0.750 |
| Postcentral gyrus (L) | 11.81 ± 2.89 | 11.16 ± 2.34 | 0.98 | 0.979 |
| Postcentral gyrus (R) | 12.66 ± 2.71 | 11.42 ± 3.04 | 1.75 | 0.774 |
| Superior parietal lobule (L) | 9.65 ± 1.95 | 9.24 ± 1.73 | 0.90 | 0.979 |
| Superior parietal lobule (R) | 9.71 ± 2.18 | 9.70 ± 1.74 | 0.03 | 0.979 |
| Inferior parietal lobule (L) | 10.17 ± 2.17 | 9.81 ± 1.50 | 0.76 | 0.979 |
| Inferior parietal lobule (R) | 10.01 ± 1.96 | 10.05 ± 1.89 | -0.07 | 0.979 |
| Supramarginal gyrus (L) | 12.53 ± 2.82 | 12.44 ± 2.92 | 0.14 | 0.979 |
| Supramarginal gyrus (R) | 12.04 ± 2.50 | 11.63 ± 1.75 | 0.72 | 0.979 |
| Angular gyrus (L) | 11.33 ± 2.18 | 10.60 ± 2.14 | 1.35 | 0.979 |
| Angular gyrus (R) | 10.43 ± 1.90 | 10.23 ± 2.30 | 0.39 | 0.979 |
| Precuneus (L) | 9.35 ± 1.76 | 9.37 ± 1.86 | -0.06 | 0.979 |
| Precuneus (R) | 9.11 ± 1.86 | 9.40 ± 1.76 | -0.63 | 0.979 |
| Paracentral lobule (L) | 9.43 ± 3.29 | 8.77 ± 2.44 | 0.89 | 0.979 |
| Paracentral lobule (R) | 9.50 ± 2.94 | 9.09 ± 2.46 | 0.60 | 0.979 |
| Caudate (L) | 9.94 ± 3.12 | 9.80 ± 1.98 | 0.20 | 0.979 |
| Caudate (R) | 10.07 ± 3.44 | 9.52 ± 2.88 | 0.69 | 0.979 |
| Putamen (L) | 15.91 ± 3.11 | 15.81 ± 3.06 | 0.13 | 0.979 |
| Putamen (R) | 16.39 ± 2.92 | 16.45 ± 3.16 | -0.09 | 0.979 |
| Pallidum (L) | 14.59 ± 3.52 | 13.76 ± 2.51 | 1.05 | 0.979 |
| Pallidum (R) | 15.42 ± 3.17 | 15.15 ± 2.57 | 0.36 | 0.979 |
| Thalamus (L) | 10.79 ± 3.22 | 10.71 ± 2.61 | 0.11 | 0.979 |
| Thalamus (R) | 10.68 ± 3.14 | 11.22 ± 2.66 | -0.74 | 0.979 |
| Heschl's gyrus (L) | 15.11 ± 3.46 | 15.19 ± 3.16 | -0.10 | 0.979 |
| Heschl's gyrus (R) | 15.81 ± 3.48 | 14.91 ± 3.43 | 1.04 | 0.979 |
| Superior temporal gyrus (L) | 16.81 ± 3.42 | 16.31 ± 3.36 | 0.59 | 0.979 |
| Superior temporal gyrus (R) | 16.30 ± 3.41 | 15.68 ± 3.62 | 0.72 | 0.979 |
| Temporal pole (superior) (L) | 16.89 ± 3.39 | 16.67 ± 3.50 | 0.27 | 0.979 |
| Temporal pole (superior) (R) | 17.01 ± 3.23 | 17.26 ± 3.11 | -0.32 | 0.979 |
| Middle temporal gyrus (L) | 14.17 ± 3.68 | 12.42 ± 3.23 | 2.00 | 0.750 |
| Middle temporal gyrus (R) | 13.24 ± 3.50 | 11.64 ± 3.16 | 1.92 | 0.750 |
| Temporal pole (middle) (L) | 13.95 ± 3.25 | 12.57 ± 2.62 | 1.83 | 0.750 |
| Temporal pole (middle) (R) | 14.36 ± 2.68 | 12.66 ± 2.41 | 2.65 | 0.450 |
| Inferior temporal gyrus (L) | 10.85 ± 2.56 | 10.70 ± 2.18 | 0.24 | 0.979 |
| Inferior temporal gyrus (R) | 11.88 ± 3.28 | 11.31 ± 2.83 | 0.74 | 0.979 |

Mean and SD were acquired from the principal dataset.

Abbreviation: ACC, anterior cingulate cortex; ADHD, attention-deficit/hyperactivity disorder; L, left; mADHD, mild symptom ADHD; PFC, prefrontal cortex; R, right; sADHD, severe symptom ADHD; SD, standard deviation; TDC, typically developing controls.
