# Supplementary material for: Novel subgroups of attention-deficit/hyperactivity disorder identified by topological data analysis and their functional network modular organizations
Source: PLoS One. 2017 Aug 22;12(8):e0182603. doi: 10.1371/journal.pone.0182603 (PMC5567504; doi:10.1371/journal.pone.0182603)
Supplement: S9 Table — (DOCX) [file pone.0182603.s011.docx]

**S9 Table**. Mean values of between centrality for each inattentive and combined subtype and its statistical comparison using two-sample t-test

| Anatomical Region | Inattentive type | Combined type | *T* | Corrected *P* |
| --- | --- | --- | --- | --- |
|  | Mean ± SD | Mean ± SD |  |  |
| Precentral gyrus (L) | 0.033 ± 0.045 | 0.040 ± 0.048 | -0.59 | 0.886 |
| Precentral gyrus (R) | 0.040 ± 0.047 | 0.030 ± 0.030 | 0.94 | 0.875 |
| Superior frontal gyrus (L) | 0.042 ± 0.049 | 0.034 ± 0.043 | 0.62 | 0.886 |
| Superior frontal gyrus (R) | 0.041 ± 0.055 | 0.039 ± 0.045 | 0.15 | 0.979 |
| Orbitofrontal cortex (superior) (L) | 0.051 ± 0.078 | 0.025 ± 0.026 | 1.66 | 0.821 |
| Orbitofrontal cortex (superior) (R) | 0.037 ± 0.037 | 0.045 ± 0.055 | -0.78 | 0.875 |
| Dorsolateral PFC (L) | 0.038 ± 0.069 | 0.047 ± 0.069 | -0.53 | 0.886 |
| Dorsolateral PFC (R) | 0.024 ± 0.035 | 0.051 ± 0.082 | -1.85 | 0.690 |
| Orbitofrontal cortex (middle) (L) | 0.046 ± 0.052 | 0.051 ± 0.074 | -0.30 | 0.958 |
| Orbitofrontal cortex (middle) (R) | 0.033 ± 0.039 | 0.055 ± 0.057 | -1.85 | 0.690 |
| Inferior frontal gyrus (operculuar) (L) | 0.041 ± 0.047 | 0.038 ± 0.047 | 0.25 | 0.979 |
| Inferior frontal gyrus (opercular) (R) | 0.032 ± 0.045 | 0.031 ± 0.028 | 0.11 | 0.979 |
| Inferior frontal gyrus (triangular) (L) | 0.029 ± 0.034 | 0.032 ± 0.030 | -0.41 | 0.895 |
| Inferior frontal gyrus (triangular) (R) | 0.035 ± 0.046 | 0.026 ± 0.028 | 0.92 | 0.875 |
| Inferior frontal gyrus (orbitalis) (L) | 0.040 ± 0.055 | 0.025 ± 0.032 | 1.34 | 0.828 |
| Inferior frontal gyrus (orbitalis) (R) | 0.024 ± 0.033 | 0.017 ± 0.022 | 1.03 | 0.875 |
| Rolandic operculum (L) | 0.027 ± 0.042 | 0.024 ± 0.045 | 0.23 | 0.979 |
| Rolandic operculum (R) | 0.041 ± 0.062 | 0.026 ± 0.033 | 1.17 | 0.875 |
| Supplementary motor area (L) | 0.047 ± 0.062 | 0.029 ± 0.036 | 1.35 | 0.828 |
| Supplementary motor area (R) | 0.033 ± 0.040 | 0.043 ± 0.046 | -0.87 | 0.875 |
| Olfactory (L) | 0.040 ± 0.053 | 0.053 ± 0.068 | -0.87 | 0.875 |
| Olfactory (R) | 0.048 ± 0.054 | 0.042 ± 0.053 | 0.47 | 0.886 |
| Dorsomedial PFC (L) | 0.030 ± 0.042 | 0.036 ± 0.048 | -0.51 | 0.886 |
| Dorsomedial PFC (R) | 0.049 ± 0.071 | 0.049 ± 0.061 | 0.04 | 0.983 |
| Ventromedial PFC (L) | 0.040 ± 0.047 | 0.038 ± 0.059 | 0.14 | 0.979 |
| Ventromedial PFC (R) | 0.026 ± 0.041 | 0.031 ± 0.046 | -0.46 | 0.886 |
| Rectus gyrus (L) | 0.037 ± 0.038 | 0.049 ± 0.061 | -1.07 | 0.875 |
| Rectus gyrus (R) | 0.036 ± 0.036 | 0.040 ± 0.042 | -0.46 | 0.886 |
| Insula (L) | 0.022 ± 0.038 | 0.013 ± 0.019 | 1.17 | 0.875 |
| Insula (R) | 0.024 ± 0.034 | 0.017 ± 0.024 | 0.84 | 0.875 |
| Ventral ACC (L) | 0.032 ± 0.046 | 0.041 ± 0.083 | -0.56 | 0.886 |
| Ventral ACC (R) | 0.019 ± 0.029 | 0.019 ± 0.036 | -0.04 | 0.983 |
| Dorsal ACC (L) | 0.056 ± 0.057 | 0.056 ± 0.061 | 0.00 | 0.997 |
| Dorsal ACC (R) | 0.041 ± 0.050 | 0.053 ± 0.062 | -0.87 | 0.875 |
| Posterior cingulate cortex (L) | 0.032 ± 0.038 | 0.037 ± 0.043 | -0.49 | 0.886 |
| Posterior cingulate cortex (R) | 0.024 ± 0.032 | 0.033 ± 0.034 | -1.16 | 0.875 |
| Hippocampus (L) | 0.042 ± 0.056 | 0.041 ± 0.044 | 0.06 | 0.983 |
| Hippocampus (R) | 0.044 ± 0.057 | 0.031 ± 0.039 | 1.06 | 0.875 |
| Parahippocampal gyrus (L) | 0.047 ± 0.051 | 0.030 ± 0.044 | 1.41 | 0.828 |
| Parahippocampal gyrus (R) | 0.060 ± 0.059 | 0.023 ± 0.026 | 3.12 | 0.135 |
| Amygdala (L) | 0.025 ± 0.038 | 0.037 ± 0.059 | -1.02 | 0.875 |
| Amygdala (R) | 0.038 ± 0.049 | 0.022 ± 0.026 | 1.55 | 0.821 |
| Calcarine cortex (L) | 0.031 ± 0.038 | 0.030 ± 0.037 | 0.12 | 0.979 |
| Calcarine cortex (R) | 0.047 ± 0.057 | 0.054 ± 0.070 | -0.47 | 0.886 |
| Cuneus (L) | 0.024 ± 0.031 | 0.025 ± 0.039 | -0.10 | 0.979 |
| Cuneus (R) | 0.027 ± 0.035 | 0.029 ± 0.038 | -0.23 | 0.979 |
| Lingual gyrus (L) | 0.035 ± 0.040 | 0.045 ± 0.054 | -0.82 | 0.875 |
| Lingual gyrus (R) | 0.028 ± 0.040 | 0.040 ± 0.036 | -1.20 | 0.875 |
| Superior occipital gyrus (L) | 0.032 ± 0.037 | 0.023 ± 0.033 | 1.02 | 0.875 |
| Superior occipital gyrus (R) | 0.040 ± 0.067 | 0.019 ± 0.029 | 1.54 | 0.821 |
| Middle occipital gyrus (L) | 0.033 ± 0.045 | 0.025 ± 0.037 | 0.78 | 0.875 |
| Middle occipital gyrus (R) | 0.040 ± 0.057 | 0.023 ± 0.040 | 1.37 | 0.828 |
| Inferior occipital gyrus (L) | 0.045 ± 0.051 | 0.029 ± 0.030 | 1.47 | 0.821 |
| Inferior occipital gyrus (R) | 0.024 ± 0.025 | 0.044 ± 0.048 | -2.28 | 0.690 |
| Fusiform gyrus (L) | 0.047 ± 0.069 | 0.034 ± 0.040 | 0.92 | 0.875 |
| Fusiform gyrus (R) | 0.035 ± 0.042 | 0.043 ± 0.050 | -0.69 | 0.886 |
| Postcentral gyrus (L) | 0.031 ± 0.038 | 0.019 ± 0.019 | 1.55 | 0.821 |
| Postcentral gyrus (R) | 0.025 ± 0.033 | 0.028 ± 0.026 | -0.42 | 0.895 |
| Superior parietal lobule (L) | 0.035 ± 0.057 | 0.026 ± 0.030 | 0.75 | 0.877 |
| Superior parietal lobule (R) | 0.029 ± 0.040 | 0.028 ± 0.033 | 0.18 | 0.979 |
| Inferior parietal lobule (L) | 0.029 ± 0.039 | 0.034 ± 0.045 | -0.46 | 0.886 |
| Inferior parietal lobule (R) | 0.053 ± 0.050 | 0.049 ± 0.049 | 0.37 | 0.918 |
| Supramarginal gyrus (L) | 0.038 ± 0.057 | 0.030 ± 0.037 | 0.59 | 0.886 |
| Supramarginal gyrus (R) | 0.023 ± 0.029 | 0.043 ± 0.048 | -2.15 | 0.690 |
| Angular gyrus (L) | 0.025 ± 0.028 | 0.039 ± 0.045 | -1.53 | 0.821 |
| Angular gyrus (R) | 0.044 ± 0.044 | 0.035 ± 0.055 | 0.73 | 0.881 |
| Precuneus (L) | 0.024 ± 0.035 | 0.017 ± 0.033 | 0.81 | 0.875 |
| Precuneus (R) | 0.019 ± 0.022 | 0.016 ± 0.027 | 0.48 | 0.886 |
| Paracentral lobule (L) | 0.040 ± 0.047 | 0.061 ± 0.068 | -1.47 | 0.821 |
| Paracentral lobule (R) | 0.033 ± 0.041 | 0.046 ± 0.062 | -0.98 | 0.875 |
| Caudate (L) | 0.032 ± 0.045 | 0.031 ± 0.037 | 0.12 | 0.979 |
| Caudate (R) | 0.026 ± 0.036 | 0.046 ± 0.041 | -2.09 | 0.690 |
| Putamen (L) | 0.034 ± 0.056 | 0.027 ± 0.039 | 0.54 | 0.886 |
| Putamen (R) | 0.029 ± 0.044 | 0.027 ± 0.055 | 0.21 | 0.979 |
| Pallidum (L) | 0.018 ± 0.028 | 0.054 ± 0.054 | -3.54 | 0.090 |
| Pallidum (R) | 0.033 ± 0.040 | 0.036 ± 0.042 | -0.34 | 0.932 |
| Thalamus (L) | 0.033 ± 0.047 | 0.025 ± 0.034 | 0.77 | 0.875 |
| Thalamus (R) | 0.044 ± 0.054 | 0.043 ± 0.079 | 0.10 | 0.979 |
| Heschl's gyrus (L) | 0.028 ± 0.045 | 0.032 ± 0.037 | -0.43 | 0.895 |
| Heschl's gyrus (R) | 0.046 ± 0.059 | 0.033 ± 0.049 | 0.93 | 0.875 |
| Superior temporal gyrus (L) | 0.022 ± 0.025 | 0.032 ± 0.040 | -1.23 | 0.875 |
| Superior temporal gyrus (R) | 0.032 ± 0.048 | 0.045 ± 0.054 | -1.03 | 0.875 |
| Temporal pole (superior) (L) | 0.020 ± 0.027 | 0.037 ± 0.048 | -1.88 | 0.690 |
| Temporal pole (superior) (R) | 0.030 ± 0.049 | 0.019 ± 0.029 | 1.00 | 0.875 |
| Middle temporal gyrus (L) | 0.035 ± 0.061 | 0.043 ± 0.047 | -0.59 | 0.886 |
| Middle temporal gyrus (R) | 0.036 ± 0.045 | 0.041 ± 0.051 | -0.46 | 0.886 |
| Temporal pole (middle) (L) | 0.045 ± 0.060 | 0.044 ± 0.050 | 0.06 | 0.983 |
| Temporal pole (middle) (R) | 0.045 ± 0.050 | 0.053 ± 0.067 | -0.55 | 0.886 |
| Inferior temporal gyrus (L) | 0.034 ± 0.041 | 0.036 ± 0.043 | -0.13 | 0.979 |
| Inferior temporal gyrus (R) | 0.055 ± 0.065 | 0.028 ± 0.048 | 1.85 | 0.690 |

Mean and SD were acquired from the principal dataset.

Abbreviation: ACC, anterior cingulate cortex; ADHD, attention-deficit/hyperactivity disorder; L, left; mADHD, mild symptom ADHD; PFC, prefrontal cortex; R, right; sADHD, severe symptom ADHD; SD, standard deviation; TDC, typically developing controls.
