# Supplementary material for: Novel subgroups of attention-deficit/hyperactivity disorder identified by topological data analysis and their functional network modular organizations
Source: PLoS One. 2017 Aug 22;12(8):e0182603. doi: 10.1371/journal.pone.0182603 (PMC5567504; doi:10.1371/journal.pone.0182603)
Supplement: S10 Table — (DOCX) [file pone.0182603.s012.docx]

**S10 Table**. Mean values of PageRank centrality for each inattentive and combined subtype and its statistical comparison using two-sample t-test

| Anatomical Region | Inattentive type | Combined type | *T* | Corrected *P* |
| --- | --- | --- | --- | --- |
|  | Mean ± SD | Mean ± SD |  |  |
| Precentral gyrus (L) | 0.0096 ± 0.0019 | 0.0091 ± 0.0013 | 1.10 | 0.888 |
| Precentral gyrus (R) | 0.0108 ± 0.0015 | 0.0109 ± 0.0017 | -0.37 | 0.987 |
| Superior frontal gyrus (L) | 0.0102 ± 0.0016 | 0.0100 ± 0.0018 | 0.34 | 0.987 |
| Superior frontal gyrus (R) | 0.0094 ± 0.0015 | 0.0099 ± 0.0020 | -1.34 | 0.799 |
| Orbitofrontal cortex (superior) (L) | 0.0118 ± 0.0017 | 0.0118 ± 0.0019 | 0.17 | 0.987 |
| Orbitofrontal cortex (superior) (R) | 0.0107 ± 0.0017 | 0.0111 ± 0.0018 | -0.95 | 0.888 |
| Dorsolateral PFC (L) | 0.0094 ± 0.0015 | 0.0099 ± 0.0015 | -1.52 | 0.704 |
| Dorsolateral PFC (R) | 0.0094 ± 0.0014 | 0.0098 ± 0.0016 | -1.26 | 0.799 |
| Orbitofrontal cortex (middle) (L) | 0.0102 ± 0.0015 | 0.0113 ± 0.0017 | -2.75 | 0.300 |
| Orbitofrontal cortex (middle) (R) | 0.0104 ± 0.0014 | 0.0108 ± 0.0018 | -1.05 | 0.888 |
| Inferior frontal gyrus (operculuar) (L) | 0.0115 ± 0.0017 | 0.0114 ± 0.0015 | 0.29 | 0.987 |
| Inferior frontal gyrus (opercular) (R) | 0.0117 ± 0.0016 | 0.0117 ± 0.0013 | 0.02 | 0.987 |
| Inferior frontal gyrus (triangular) (L) | 0.0111 ± 0.0016 | 0.0111 ± 0.0016 | -0.11 | 0.987 |
| Inferior frontal gyrus (triangular) (R) | 0.0110 ± 0.0015 | 0.0116 ± 0.0015 | -1.60 | 0.681 |
| Inferior frontal gyrus (orbitalis) (L) | 0.0122 ± 0.0018 | 0.0126 ± 0.0018 | -0.81 | 0.987 |
| Inferior frontal gyrus (orbitalis) (R) | 0.0130 ± 0.0015 | 0.0134 ± 0.0017 | -1.04 | 0.888 |
| Rolandic operculum (L) | 0.0131 ± 0.0015 | 0.0131 ± 0.0016 | -0.02 | 0.987 |
| Rolandic operculum (R) | 0.0128 ± 0.0016 | 0.0126 ± 0.0016 | 0.34 | 0.987 |
| Supplementary motor area (L) | 0.0100 ± 0.0017 | 0.0108 ± 0.0015 | -1.86 | 0.478 |
| Supplementary motor area (R) | 0.0101 ± 0.0017 | 0.0102 ± 0.0011 | -0.25 | 0.987 |
| Olfactory (L) | 0.0108 ± 0.0016 | 0.0111 ± 0.0018 | -0.52 | 0.987 |
| Olfactory (R) | 0.0099 ± 0.0017 | 0.0101 ± 0.0018 | -0.50 | 0.987 |
| Dorsomedial PFC (L) | 0.0114 ± 0.0019 | 0.0114 ± 0.0016 | 0.05 | 0.987 |
| Dorsomedial PFC (R) | 0.0111 ± 0.0015 | 0.0112 ± 0.0016 | -0.06 | 0.987 |
| Ventromedial PFC (L) | 0.0128 ± 0.0013 | 0.0127 ± 0.0013 | 0.30 | 0.987 |
| Ventromedial PFC (R) | 0.0125 ± 0.0014 | 0.0124 ± 0.0015 | 0.12 | 0.987 |
| Rectus gyrus (L) | 0.0128 ± 0.0017 | 0.0126 ± 0.0013 | 0.41 | 0.987 |
| Rectus gyrus (R) | 0.0127 ± 0.0017 | 0.0124 ± 0.0015 | 0.67 | 0.987 |
| Insula (L) | 0.0129 ± 0.0011 | 0.0133 ± 0.0013 | -1.48 | 0.725 |
| Insula (R) | 0.0142 ± 0.0012 | 0.0143 ± 0.0011 | -0.49 | 0.987 |
| Ventral ACC (L) | 0.0120 ± 0.0015 | 0.0129 ± 0.0013 | -2.53 | 0.315 |
| Ventral ACC (R) | 0.0120 ± 0.0014 | 0.0127 ± 0.0014 | -2.20 | 0.399 |
| Dorsal ACC (L) | 0.0099 ± 0.0015 | 0.0100 ± 0.0017 | -0.41 | 0.987 |
| Dorsal ACC (R) | 0.0101 ± 0.0016 | 0.0101 ± 0.0012 | -0.08 | 0.987 |
| Posterior cingulate cortex (L) | 0.0109 ± 0.0013 | 0.0100 ± 0.0011 | 2.67 | 0.300 |
| Posterior cingulate cortex (R) | 0.0093 ± 0.0013 | 0.0086 ± 0.0014 | 2.10 | 0.420 |
| Hippocampus (L) | 0.0096 ± 0.0020 | 0.0091 ± 0.0014 | 1.24 | 0.799 |
| Hippocampus (R) | 0.0089 ± 0.0018 | 0.0091 ± 0.0014 | -0.46 | 0.987 |
| Parahippocampal gyrus (L) | 0.0099 ± 0.0021 | 0.0100 ± 0.0016 | -0.18 | 0.987 |
| Parahippocampal gyrus (R) | 0.0109 ± 0.0019 | 0.0111 ± 0.0014 | -0.40 | 0.987 |
| Amygdala (L) | 0.0125 ± 0.0020 | 0.0126 ± 0.0016 | -0.20 | 0.987 |
| Amygdala (R) | 0.0124 ± 0.0020 | 0.0130 ± 0.0017 | -1.31 | 0.799 |
| Calcarine cortex (L) | 0.0098 ± 0.0015 | 0.0103 ± 0.0012 | -1.57 | 0.681 |
| Calcarine cortex (R) | 0.0104 ± 0.0016 | 0.0109 ± 0.0013 | -1.17 | 0.827 |
| Cuneus (L) | 0.0104 ± 0.0010 | 0.0107 ± 0.0011 | -1.24 | 0.799 |
| Cuneus (R) | 0.0109 ± 0.0012 | 0.0110 ± 0.0015 | -0.29 | 0.987 |
| Lingual gyrus (L) | 0.0111 ± 0.0018 | 0.0111 ± 0.0014 | -0.04 | 0.987 |
| Lingual gyrus (R) | 0.0113 ± 0.0018 | 0.0110 ± 0.0013 | 0.56 | 0.987 |
| Superior occipital gyrus (L) | 0.0111 ± 0.0011 | 0.0111 ± 0.0013 | 0.22 | 0.987 |
| Superior occipital gyrus (R) | 0.0102 ± 0.0014 | 0.0102 ± 0.0010 | -0.16 | 0.987 |
| Middle occipital gyrus (L) | 0.0110 ± 0.0012 | 0.0114 ± 0.0011 | -1.34 | 0.799 |
| Middle occipital gyrus (R) | 0.0105 ± 0.0014 | 0.0112 ± 0.0012 | -2.25 | 0.399 |
| Inferior occipital gyrus (L) | 0.0104 ± 0.0014 | 0.0105 ± 0.0012 | -0.28 | 0.987 |
| Inferior occipital gyrus (R) | 0.0103 ± 0.0015 | 0.0099 ± 0.0013 | 1.23 | 0.799 |
| Fusiform gyrus (L) | 0.0114 ± 0.0017 | 0.0106 ± 0.0017 | 1.85 | 0.478 |
| Fusiform gyrus (R) | 0.0111 ± 0.0015 | 0.0103 ± 0.0015 | 2.21 | 0.399 |
| Postcentral gyrus (L) | 0.0107 ± 0.0019 | 0.0103 ± 0.0016 | 0.73 | 0.987 |
| Postcentral gyrus (R) | 0.0114 ± 0.0017 | 0.0106 ± 0.0020 | 1.63 | 0.681 |
| Superior parietal lobule (L) | 0.0095 ± 0.0012 | 0.0093 ± 0.0015 | 0.46 | 0.987 |
| Superior parietal lobule (R) | 0.0097 ± 0.0014 | 0.0097 ± 0.0013 | -0.10 | 0.987 |
| Inferior parietal lobule (L) | 0.0098 ± 0.0014 | 0.0096 ± 0.0011 | 0.41 | 0.987 |
| Inferior parietal lobule (R) | 0.0097 ± 0.0012 | 0.0099 ± 0.0014 | -0.44 | 0.987 |
| Supramarginal gyrus (L) | 0.0112 ± 0.0017 | 0.0113 ± 0.0017 | -0.27 | 0.987 |
| Supramarginal gyrus (R) | 0.0108 ± 0.0014 | 0.0107 ± 0.0012 | 0.16 | 0.987 |
| Angular gyrus (L) | 0.0105 ± 0.0015 | 0.0102 ± 0.0016 | 0.69 | 0.987 |
| Angular gyrus (R) | 0.0103 ± 0.0015 | 0.0103 ± 0.0017 | 0.12 | 0.987 |
| Precuneus (L) | 0.0095 ± 0.0011 | 0.0096 ± 0.0014 | -0.43 | 0.987 |
| Precuneus (R) | 0.0094 ± 0.0012 | 0.0097 ± 0.0013 | -0.93 | 0.888 |
| Paracentral lobule (L) | 0.0092 ± 0.0020 | 0.0088 ± 0.0016 | 1.00 | 0.888 |
| Paracentral lobule (R) | 0.0093 ± 0.0017 | 0.0090 ± 0.0014 | 0.93 | 0.888 |
| Caudate (L) | 0.0095 ± 0.0019 | 0.0094 ± 0.0013 | 0.31 | 0.987 |
| Caudate (R) | 0.0096 ± 0.0021 | 0.0092 ± 0.0018 | 0.79 | 0.987 |
| Putamen (L) | 0.0135 ± 0.0016 | 0.0135 ± 0.0015 | -0.03 | 0.987 |
| Putamen (R) | 0.0137 ± 0.0016 | 0.0138 ± 0.0016 | -0.29 | 0.987 |
| Pallidum (L) | 0.0126 ± 0.0019 | 0.0121 ± 0.0016 | 1.18 | 0.827 |
| Pallidum (R) | 0.0131 ± 0.0016 | 0.0130 ± 0.0013 | 0.20 | 0.987 |
| Thalamus (L) | 0.0098 ± 0.0021 | 0.0097 ± 0.0015 | 0.22 | 0.987 |
| Thalamus (R) | 0.0100 ± 0.0018 | 0.0104 ± 0.0016 | -0.97 | 0.888 |
| Heschl's gyrus (L) | 0.0126 ± 0.0019 | 0.0129 ± 0.0016 | -0.57 | 0.987 |
| Heschl's gyrus (R) | 0.0132 ± 0.0018 | 0.0128 ± 0.0019 | 0.95 | 0.888 |
| Superior temporal gyrus (L) | 0.0138 ± 0.0017 | 0.0136 ± 0.0017 | 0.38 | 0.987 |
| Superior temporal gyrus (R) | 0.0134 ± 0.0017 | 0.0132 ± 0.0020 | 0.50 | 0.987 |
| Temporal pole (superior) (L) | 0.0139 ± 0.0018 | 0.0139 ± 0.0018 | 0.02 | 0.987 |
| Temporal pole (superior) (R) | 0.0141 ± 0.0017 | 0.0144 ± 0.0017 | -0.82 | 0.987 |
| Middle temporal gyrus (L) | 0.0122 ± 0.0020 | 0.0112 ± 0.0020 | 2.07 | 0.420 |
| Middle temporal gyrus (R) | 0.0115 ± 0.0018 | 0.0107 ± 0.0019 | 1.85 | 0.478 |
| Temporal pole (middle) (L) | 0.0121 ± 0.0020 | 0.0112 ± 0.0015 | 1.98 | 0.468 |
| Temporal pole (middle) (R) | 0.0122 ± 0.0017 | 0.0111 ± 0.0013 | 2.80 | 0.300 |
| Inferior temporal gyrus (L) | 0.0100 ± 0.0018 | 0.0101 ± 0.0013 | -0.25 | 0.987 |
| Inferior temporal gyrus (R) | 0.0106 ± 0.0017 | 0.0105 ± 0.0017 | 0.36 | 0.987 |

Mean and SD were acquired from the principal dataset.

Abbreviation: ACC, anterior cingulate cortex; ADHD, attention-deficit/hyperactivity disorder; L, left; mADHD, mild symptom ADHD; PFC, prefrontal cortex; R, right; sADHD, severe symptom ADHD; SD, standard deviation; TDC, typically developing controls.
